# Prenatal maternal antibiotic use increases the risk of childhood eczema: a systematic review and meta-analysis

**DOI:** 10.1038/s41598-026-51441-x

**Published:** 2026-05-08

**Authors:** Szilárd Petrás, Bettina Vargáné Szabó, Tivadar Kiss, Muh.Akbar Bahar, Dezső Csupor, Barbara Tóth

**Affiliations:** 1 Institute of Clinical Pharmacy, Faculty of Pharmacy, University of Szeged, Szikra u. 6. Szeged, H-6725 Szeged, Hungary; 2 Institute of Pharmacognosy, Faculty of Pharmacy, University of Szeged, Szeged, Hungary; 3 Department of Pharmacy, Faculty of Pharmacy, Universitas Hasanuddin, Makassar, Indonesia; 4 Institute of Translational Medicine, University of Pécs, Pécs, Hungary

**Keywords:** Antibiotics, Atopic dermatitis, Meta-analysis, Prenatal, Systematic review, Diseases, Health care, Medical research, Microbiology, Risk factors

## Abstract

Early-life disruption of microbiome development is known to impair health; however, the long-term effects of pregnancy-related pharmacological alterations to the maternal gut microbiota on offspring health remain unclear. This meta-analysis aimed to evaluate the impact of maternal antibiotic use (either prenatal or intrapartum) on the risk of childhood atopic dermatitis, based on cohort and case-control studies. Literature searches were conducted in EMBASE, PubMed, Cochrane, and Web of Science databases using predefined PICO (patients, intervention, comparison, outcome) criteria. Overall, our meta-analysis included 30 studies with a total of 4,125,143 mothers and 4,346,050 children. Using the random effects model, our study found that prenatal antibiotic use was associated with higher odds of atopic dermatitis in childhood (aOR: 1.32; 95% CI: 1.12; 1.56). This result remained significant after adjusting publication bias by the trim-and-fill method (aOR: 1.22; 95% CI: 1.03; 1.44), highlighting the potential relevance of antibiotic prescribing practices during pregnancy in relation to childhood atopic disease risk. Intrapartum antibiotic use was not associated with elevated risk for atopic dermatitis in the children (OR: 1.64; 95% CI: 0.84; 3.17). Prenatal antibiotic use appears to have a modest effect on atopic dermatitis in offspring.

**Supplementary Information:**

The online version contains supplementary material available at 10.1038/s41598-026-51441-x.

## Introduction

It is well established that the first 1000 days of life, from conception to approximately 2 years of age, have a major influence on overall health^1^. There is a growing body of evidence that medical products considered safe in the general population may nonetheless affect the health of pregnant women and their offspring^2^. Pregnant women are more susceptible to certain types of infections, possibly due to the immunological changes that occur during gestation^3^. Therefore, it is not surprising that antibiotic prescriptions and use are relatively common among expectant women^3^. Based on a meta-analysis involving approximately 34 million pregnancies, the pooled prevalence of prenatal antibiotic use was estimated to be 23.6%^4^.

Antibiotic overuse is linked to several unfavourable health consequences, such as type 2 diabetes, autoimmune and inflammatory bowel diseases, and most importantly antimicrobial resistance (AMR)^5^. An estimated 4.95 million deaths worldwide were caused by antimicrobial resistance in 2019, making it a leading cause of death^6^. In addition to AMR, it is well-established that antibiotics disrupt the gut microbiome. The integrity of the microbiome is essential for overall health, particularly for the proper functioning of the immune system^5,7^. Based on extensive research focusing on the microbiome, there is substantial evidence suggesting that interrupting the healthy development of the microbiome at an early stage of life is detrimental tothe health of the child^8,9^. Early disruption of the gut microbiota may increase the likelihood of developing allergic diseases^8,9^, obesity or autism spectrum disorder^9^. Similar concerns have been raised regarding maternal dysbiosis and the prenatal use of medicines that alter the maternal microbiome [10 The development of the infant gut microbiome is influenced by various other factors as well, such as the mode of delivery and breastfeeding^7^.

Alterations in microbiome composition are the subject of increasingly intensive investigation. However, relatively little is known about the long-term effects of agents that disrupt microbiome^11,12^. Antibiotics are the agents with the greatest impact on the microbiome. In particular, antibiotic exposure can disturb the balance of the vaginal microbiota, which plays an important role in shaping the newborn’s gut microbiota over the long term^13^. Recent studies have linked several diseases to dysbiosis and it has also been reported that the use of antibiotics during pregnancy also increases the risk of allergic disease in early childhood^5^.

Atopic dermatitis (AD) is a chronic inflammatory skin disease, characterised by red, dry and itchy skin, which affects about 20% of children, and up to 10% of adults^14^. Due to a defective skin barrier, leading to significant water loss, and better penetration for the irritants and allergens, causing inflammation^15^.The etiology of atopic dermatitis (AD) is multifactorial, involving a complex interplay between genetic predisposition, skin barrier dysfunction, immune dysregulation, and environmental exposures^15^.. Well-established risk factors include a family history of atopic diseases, mutations in the filaggrin gene leading to impaired skin barrier function, and environmental influences such as early-life microbial exposures, mode of delivery, and feeding practices^16^.. Increasing evidence highlights the critical role of the early-life microbiome in immune system maturation and the development of allergic diseases, including AD. Disruptions to the maternal or infant microbiome—particularly during critical developmental windows—may predispose offspring to atopic conditions. Prenatal antibiotic exposure has been proposed as one such factor, as antibiotics can alter maternal gut and vaginal microbiota, potentially affecting microbial transmission to the neonate and subsequent immune programming. This provides a biologically plausible mechanism linking maternal antibiotic use during pregnancy to an increased risk of AD in offspring.

Atopic dermatitis should not be confused with eczema, which is a broader and non-specific term^17^. However, these terms are often used as synonyms given the lack of standardised nomenclature^17^. In the past three decades, childhood AD cases increased by 4.8%, mainly occurring among children between 2 and 4 years of age^18^. It is usually diagnosed before the age of two, and the lesions can occur anywhere on the body, particularly on the face and limbs^19^. The exact cause of atopic dermatitis is unknown, but both genetic and environmental factors may be involved^19^. Even though atopic dermatitis clears spontaneously in most of the cases, the affected children are more likely to develop other atopic diseases later in life^19,20^. Atopic dermatitis can be the starting point of the atopic march, which progresses to IgE mediated food allergy, asthma and allergic rhinitis^20^. In the case of atopic dermatitis, evidence suggests that, in addition to alterations in the skin microbiome, reduced intestinal flora diversity also has a significant impact in developing the disease^7^. Accordingly, antibiotic exposure, particularly in early life, has been examined as a factor potentially associated with the occurrence of this condition^7^. However, the results of the studies addressing this topic are inconsistent, possibly due to unadjusted data and unexplored subgroup analyses^21–29^. Furthermore, a recent study conducted by Chang et al. found significant results for antibiotic use and childhood eczema, but became nonsignificant after publication bias was assessed^29^, we aimed to expand the literature search for studies and therefore gain a clearer picture on the topic. Therefore, our aim was to conduct a comprehensive systematic review and meta-analysis to evaluate whether maternal antibiotic use either during the prenatal or the intrapartum period is associated with childhood eczema as our primary outcome. No secondary outcome was considered.

## Methods

### Protocol

The meta-analysis was performed according to the PRISMA protocol, and it was registered in the International Prospective Register of Systematic Reviews (PROSPERO) on June 4 2025 (registration number CRD420251011416), where the review protocol can be found. The following PICO (patients, intervention, comparison, outcome) format was applied: P: pregnant women; I: prenatal or intrapartum antibiotic therapy; C: no antibiotic useantibiotics; and O: atopic dermatitis or eczema in the offspring.

### Information sources and search strategy

A literature search was conducted until 1 August 2025 in EMBASE, Medline (via PubMed), Cochrane Central Register of Controlled Trials (CENTRAL) and Web of Science. The search syntax was as follows: (antibiotic) AND ((Atopic dermatitis) OR Eczema). No language, publication date or publication status restrictions were applied. The reference lists of all identified articles were inspected. Only publicly available data were analysed, the authors were not contacted for additional information.

### Eligibility criteria and study selection

Studies evaluating the effects of maternal antibiotic therapy on the prevalence of atopic dermatitis or eczema in offspring were included, including cohort, cross-sectional and case-control studies. Due to the lack of standardized nomenclature, we included both outcomes. Abstracts, case series, case reports, systematic reviews, meta-analyses, editorial letters, and studies not reporting numerical data were excluded from the meta-analysis. For reference management, Zotero 7.0 was used. After removing duplicates, the remaining records were screened for eligibility based on their titles and abstracts. The eligibility of the full texts of the resulting records was assessed by two reviewers independently. Disagreements between reviewers were dissolved by discussion.

### Data extraction and synthesis of results

Data collection was executed following the PRISMA guidelines. Study characteristics and results were extracted by the two reviewers independently. The following data items were extracted from the included papers: study design, years of data collection, number of mothers and children involved, number of mothers and children at the end of the study, age at the time of diagnosis, method of data collection, number of vaginal births, number of caesarean sections, time of the diagnosis of atopic dermatitis/eczema, exposure data and timing of antibiotic use and its effect size with 95% CI. Discrepancies in extracted data were resolved by discussion between the two reviewers.

### Statistical analysis

The meta-analysis was conducted by using a random effects model, which accounts for variability between studies. In this method, the aim is to estimate the mean of a distribution of effects. The pooled effect estimates were presented with 95% confidence intervals (CIs). Odds ratios (ORs), adjusted odds ratios (aOR) and adjusted hazard ratios (aHR) were assessed separately. Odds ratios were calculated from the population data of the individual studies, and where it was not possible to extract all the required population data for OR calculation, the ORs provided by the individual studies were used. To assess statistical heterogeneity, the I^2^ statistic and the τ^2^ value were used. According to the Cochrane Handbook, I^2^ can be low (0–40%), moderate (30–60%), substantial (50–90%), or considerable heterogeneity (75–100%). We performed leave-one-out analyses for sensitivity analysis.

Forest plots were used to visually display effect sizes and confidence intervals for each study and pooled results. Data visualization was performed using R metafor package. We synthesized the results narratively when it was not possible to include a study in the meta-analysis. Furthermore, to assess the potential asymmetry between the studies and the subsequent publication bias, funnel plots were made, and Egger’s test was performed. To account for the potential publication bias, trim and fill method was used.

To further assess the heterogeneity between the studies, subgroup analysis was performed. The results were presented as OR, aOR and aHR data, with 95% CI. If the studies had data for multiple subgroups, we would include in all of them.

### Risk of bias

The Risk of Bias In Non‐Randomized Studies–of Interventions (ROBINS‐I) tool was used for assessing the risk of bias of the observational studies. After evaluation, a traffic light plot was made via robvis in which low, moderate, serious, critical risk of bias or no information was indicated with green, yellow, orange, red and blue colours respectively (see Supplementary Figure 20, 21).

### Quality of evidence

The Grading of Recommendations Assessment, Development and Evaluation (GRADE) was used for estimating the quality of evidence of all outcomes assessed^30^. Certainty of evidence was rated as high, moderate, low or very low. The assessment was conducted by two independent researchers, and any disagreements were resolved by discussion.

## Results

### Literature search

**Fig. 1:**
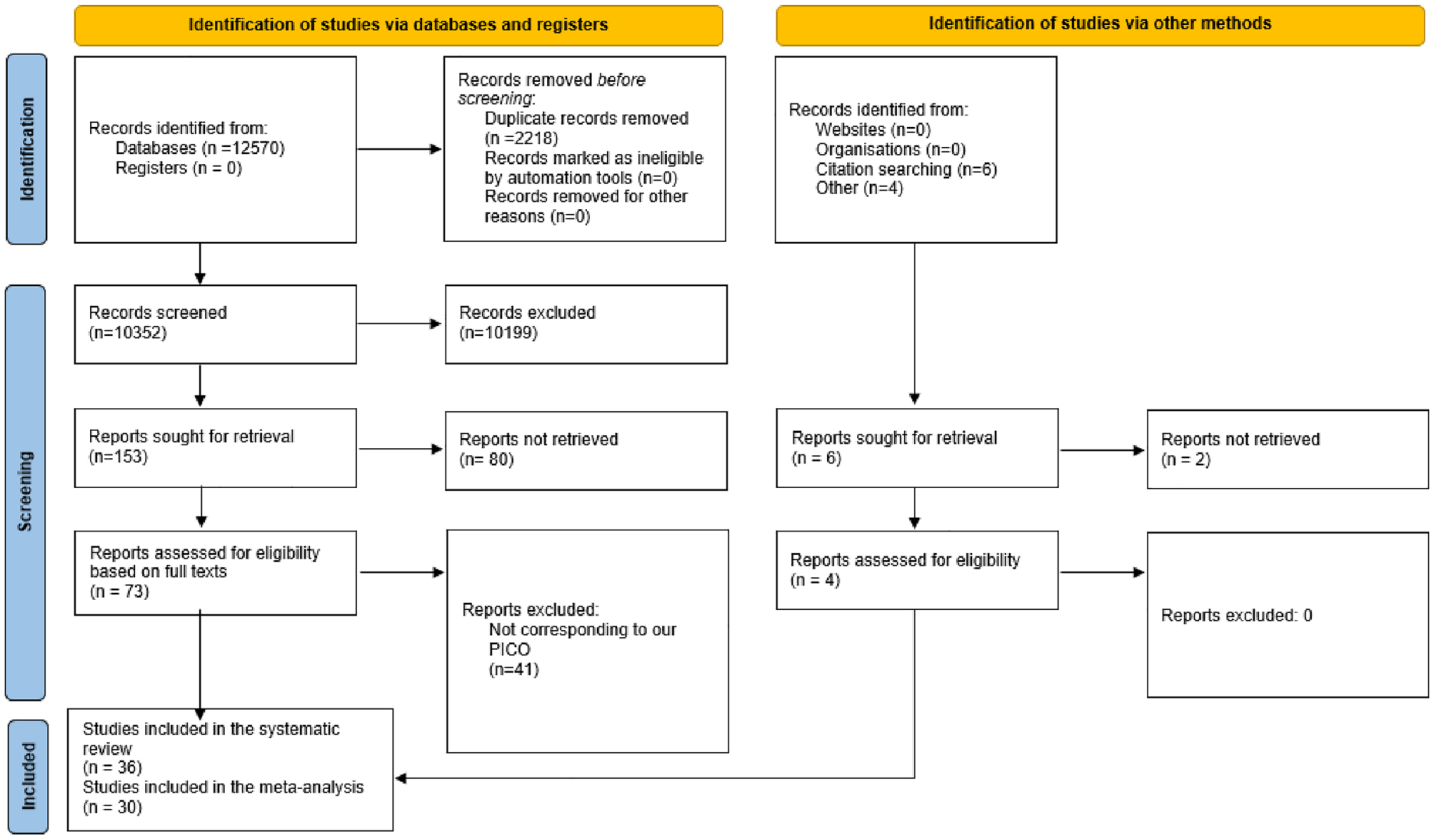
The PRISMA flow diagram for the identification of relevant studies.

Initially, a total of 12,570 records were identified from all databases based on our search strategies. After duplicate removal 10,352 studies remained, of which 10,199 were excluded based on title. An additional 10 studies were identified through other sources (citation searching of the included studies), of which 4 were included in the meta-analysis and 6 were excluded. After this, 153 studies remained for screening. Of these, 127 were excluded: 80 based on abstracts (Cohen’s kappa = 0.84), 41 based on full-text assessment (Cohen’s kappa = 0.89), and 6 identified through citation searching. The reasons for exclusion are listed in Supplementary Table 1. After assessing the full texts, 6 studies were not eligible for further statistical analysis; however, the results of these add to the broader picture of this topic; therefore, the results of these studies were briefly summarized below. A total of 30 studies were included in the statistical analysis (Figure 1).

### Qualitative analysis

A total of 36 studies complied with our PICO criteria. Of these, 30 were included in the meta-analysis and 6 studies were excluded from further statistical analysis for various reasons. In two cases only the environmental exposure to antibiotics was considered, and the studies did not have a control group^31,32^. Zheng et al. reported a higher incidence of allergy related outcomes in the high-antibiotic group, but in the case of atopic dermatitis the difference was only significant for trimethoprim exposure (OR: 2.00; 95% CI: 1.08; 3.71)^31^. Geng et al. also found an increased risk for current eczema when exposed to certain specific antibiotics prenatally in different trimesters^32^. However, the results were only significant for ciprofloxacin (aOR: 1.17; 95% CI: 1.06; 1.29) in the second trimester and enrofloxacin (aOR: 1.29; 95% CI: 1.08; 1.54) and ciprofloxacin (aOR: 1.24; 95% CI: 1.08; 1.43) in the third trimester.

We also excluded the study conducted by Batool et al. for not having a specific ‘eczema’ group, only a collective ‘allergic disease’ and ‘atopy’ group, referring to at least one positive skin prick test^33^.

We excluded two studies from the quantitative analysis for missing data. However, it is important to note that Wegienka et al. did not find a significant risk for eczema (RR: 0.81; 95% CI: 0.57; 1.15) in vaginal delivery cases^34^. Similarly, Lapin et al. found no significant associations between prenatal antibiotic use and childhood eczema (p = 0.36)^35^. We also excluded the study conducted by Sumilo et al. from the intrapartum analysis due to missing data^36^.

### Quantitative analysis

A total of 30 studies were included in the statistical analysis^37–66^. 26 of these studied the entire prenatal period, while 4 studies only examined the intrapartum period. (Table 1, 2) Of the prenatal studies included in the meta-analysis, 23 were cohort studies, one was a case-control study, one was an etiologic study based on prospective cohort, and one was a cross-sectional study. (Table 1) Seven studies were from Asia [Taiwan (3), Japan (2), China (1), Korea (1)], 15 were from Europe [Denmark (3), United Kingdom (3), Sweden (3), Belgium (2), Poland (1), Romania (1), Greece (1) and one study was conducted in multiple countries), one study was from Australia and three studies were from the United States. (Table 1) Regarding the intrapartum studies, 3 were cohort studies and one was a case-control study (Table 2). One of them was from Europe [Finland (1)], 2 were from the United States and one was from Asia (China). (Table 2) The population data of the prenatal studies are summarized in Table 1 and additional data is listed in Supplementary Table 2. The population data and results of the intrapartum studies are listed in Table 2, and additional data is found in Supplementary Table 3. Overall, our meta-analysis consisted of 4,328,170 children and 4,107,696 mothers in the prenatal group, but it is important to note that in 12 studies there was no exact data on the number of mothers involved at the end of the study and therefore were left out of the sum. In these cases, the total number of children was 217,092^37–43,52,58,60–62^. The intrapartum studies consisted of 17,880 children and 17,447 mothers; however, in the case of Puisto et al. the number of mothers at the end of the study is unknown^64^. In this case, the number of children was 433. Every included study had data on the exact number of children involved, but they varied greatly in the number of participants. All the included studies gave data on exposure and outcome assessment and the origin of diagnosis. In two cases, the outcome was assessed by clinical research units or nurses, in 9 cases data was obtained from databases in 14 cases it was parent reported and in one case databases were simultaneously used with parent reports (Supplementary Table 2). In the intrapartum group the outcome was assessed by study physicians in one case, 2 cases by database, and in one case it was parent reported. (Supplementary Table 3) The age of diagnosis also varied between the prenatal studies. In 14 cases, children were 18 months old or younger, in six cases they were older than 18 months, but not older than 5 years, and in 6 cases, they were older than 5 years. Two studies even gave data up to 18 years of age (Table 1). On the other hand, the age of diagnosis did not vary as much in the intrapartum group. In three cases it was 2 years, and in one case it was 5 years.

**Table 1:** The population data of the included prenatal studies.

Author name, year	Country	Study type	Age at the time of diagnosis	Outcome measures
Ahmad, 2021^53^	Australia	Prospective cohort	up to 15 years	OR: 1.03; 95% CI 0.79; 1.35†
Bisgaard, 2009^62^	Denmark	Prospective cohort	3 years	3rd trimester use OR: 0.65; 95% CI: 0.35; 1.22
Chang, 2023^48^	Taiwan	Retrospective cohort	0.81 ± 1.1 years^§^	OR 1.15; 95% CI 1.14; 1.16
Choi, 2025^43^	USA	Retrospective cohort	1 year	OR calculated from population data: 0.83; 95% CI: 0.80; 0.87
Dom, 2011^45^	Belgium	Aetiologic study based on prospective cohort	4 years	aOR: 1.89; 95% CI: 1.29; 2.77
El-Heis, 2023^56^	UK	Prospective cohort	1 year	pooled aOR: 1.04; 95% CI: 0.92; 1.18
Fuxench, 2024^50^	UK	Population-based cohort	10 years	OR: 1.30 95% CI: 1.29; 1.32
Gao, 2019^44^	China	Prospective cohort	1 year	OR: 3.51; 95% CI: 1.26; 9.79
Hesla, 2017^52^	Sweden	Prospective cohort	2 years	OR: 2.0; 95% CI: 1.2; 3.3
Jedrychowsky, 2006^37^	Poland	Prospective cohort	1 year	OR: 1.72; 95% CI: 0.71; 4.14
Kelderer, 2021^46^	Sweden	Prospective cohort	18 months	OR: 1.08; 95% CI: 0.72; 1.63
Kurzius-Spencer, 2004^38^	USA	Prospective cohort	1 year	OR: 0.74; 95% CI: 0.4; 1.36
Lee, 2014^58^	Korea	Prospective cohort	1 year	aOR: 5.7; 95% CI 1.19; 27.3
Lin, 2022^61^	Taiwan	Population-based nested case–control study	2.6 ± 2.9 years old^§^	OR: 1.90 95% CI: 1.78; 2.02
McKeever, 2002^57^	UK	Retrospective cohort	1.17 years (mean)	aHR: 1.17; 95% CI: 1.06; 1.29
Metzler, 2019^39^	Rural parts of Austria, Finland, France, Germany and Switzerland	Prospective cohort	up to 6 years	aOR: 1.19; 95% CI: 0.69; 2.05 ‡
Mubanga, 2021^49^	Sweden	Prospective cohort	5.8 ± 2.4 years^§^	OR: 1.14; 95% CI: 1.12; 1.16
Okoshi, 2023^59^	Japan	Prospective birth cohort	3 years	aOR: 1.02; 95% CI: 0.97; 1.08
Panduru, 2020^47^	Romania	Cross sectional study	18 years	OR: 1.28; 95% CI: 0.99; 1.65
Sariachvili, 2007^40^	Belgium	Prospective cohort	1 year	OR 1.67; 95% CI: 1.16; 2.40
Sasaki, 2019^41^	Japan	Prospective cohort	1 year	OR: 1.01; 95% CI: 0.97; 1.05
Stefanaki, 2023^54^	Greece	Prospective cohort	18 months	OR: 7.7; 95% CI: 1.23; 48.27
Stensballe, 2013^60^	Denmark	Prospective cohort	18 months	OR: 1.06; 95% CI: 0.93; 1.21
Tai, 2024^51^	Taiwan	Prospective cohort	5 years	OR: 1.16 95% CI 1.15; 1.17
Timm, 2017^55^	Denmark	Prospective cohort	18 months	pooled OR: 1.08; 95% CI: 1.02; 1.14
Vance, 2023^42^	USA	Retrospective cohort	15–22 years	OR: 1.48 95% CI: 1.25; 1.76

**Table 2:** The study data of the included intrapartum studies.

**Author name, *year***	**Country**	**Study type**	**Age at the time of diagnosis**	**Outcome measures**	**Calculated from population data**
Dhudasia, 2021^66^	USA	Retrospective cohort	5 years	For vaginal birth: aOR: 1.37; 95% CI: 0.99; 1.88 For C-section: aOR 1.39; 95% CI: 0.95; 2.04	For vaginal birth: OR: 1.28; 95% CI: 1.09; 1.51 For C-section: OR: 1.48; 95% CI: 1.14; 1.94
Hong, 2022^65^	China	Retrospective cohort	2 years	For vaginal birth: aOR: 6.56; 95% CI: 4.3; 10.0 For C-section: aOR: 0.56; 95% CI: 0.27; 1.16	For vaginal birth: OR: 6.72; 95% CI: 4.73; 9.54 For C-section: OR: 0.56; 95% CI: 0.28; 1.12
Puisto, 2022^64^	Finland	Case-control	2 years	aOR 2.21; 95% CI: 1.2; 4.1	OR: 2.26; 95% CI: 1.24; 4.12
Wohl, 2015^63^	USA	Retrospective cohort	2 years	RR: 1.03; 95% CI: 0.75; 1.41 when exposed longer than 24 hrs: RR: 1.99; 95% CI: 1.13; 3.49	OR: 1.07; 95% CI: 0.69; 1.66

### Analyses of antibiotic exposure during pregnancy and the risk of childhood atopic dermatitis

**Fig. 2:**
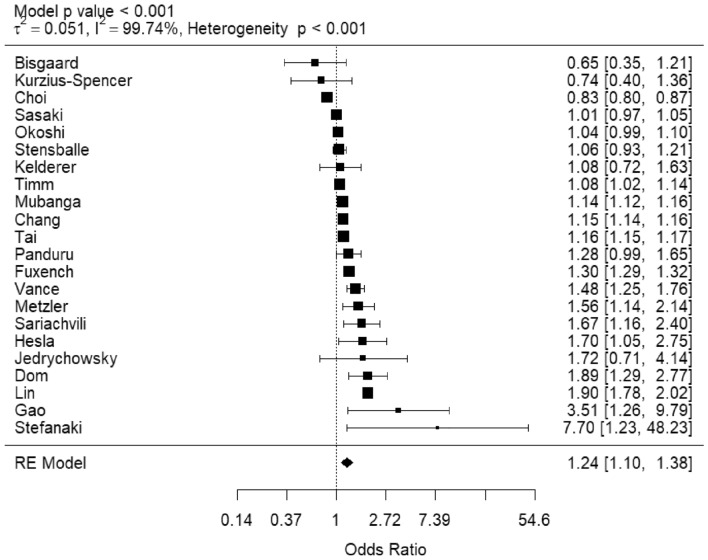
The effect of prenatal antibiotic exposure on childhood eczema based on OR data.

Of the 26 studies assessing prenatal antibiotic exposure, 22 gave OR data or had adequate population data to calculate OR from^37–52,54,55,59–62^. The overall odds ratio indicates that prenatal antibiotic exposure is associated with higher odds of atopic dermatitis in childhood (OR 1.24; 95% CI: 1.10; 1.38). (Figure 2) The studies were found to be considerably heterogenous, but the result remained significant even after the leave out one analysis (Supplementary Figure 1).

**Fig. 3:**
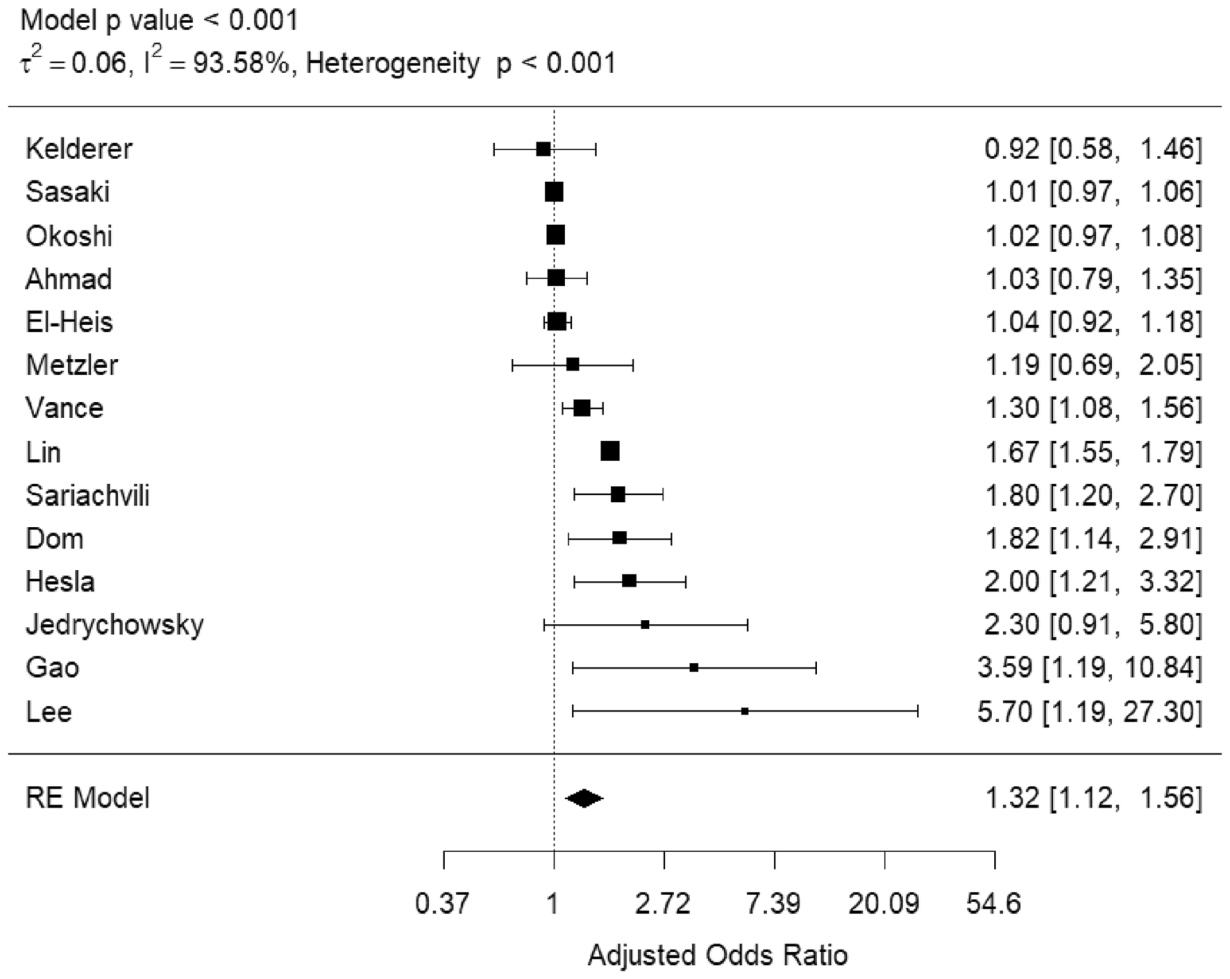
The effect of prenatal antibiotic exposure on childhood eczema based on aOR data.

Furthermore, 14 out of the 26 prenatal studies gave adjusted odds ratio data^37,39–42,44–46,52,53,56,58,59,61^. The studies adjusted for various different covariates, but the most common confounders were infant sex, maternal age, maternal allergy or atopy, and maternal smoking during pregnancy. Our meta-analysis revealed a statistically significant association between the increased risk of atopic dermatitis following prenatal exposure to antibiotics, with an adjusted odds ratio (aOR) of 1.32 95% CI: 1.12; 1.56. (Figure 3), which remained significant even after the leave-one-out analysis (Supplementary Figure 2).

To assess the potential asymmetry of the studies, funnel plots were made, which indicated significant asymmetry in both the OR cases and the aOR cases, (Supplementary Figures 3, 4) and the Egger’s test revealed significant publication bias in both cases (for OR: z=2.0749, p=0.038 and for aOR: z=2.8647, p=0.0042). To address this bias, trim-and-fill method was performed, and the results remained significant in both cases OR: 1.21; 95% CI: 1.08; 1.36; and aOR: 1.22; 95% CI: 1.03; 1.44.

Four out of the 26 prenatal studies gave aHR data for measuring the risk for prenatal antibiotic use^48,49,51,57^. Similarly, these studies also found an increased risk for developing atopic dermatitis in childhood with a pooled aHR of 1.10 (95% CI: 1.05; 1.14). The studies adjusted for various confounders, but the most prevalent were maternal age, infant’s sex and the presence of maternal atopic disorders (Supplementary Figure 5).

It is important to note that the statistical heterogeneity was high in all cases. Among the studies which gave OR data the I^2^ was 99.74%, in the aOR cases it was 93.58%, and for the aHR studies the heterogeneity was 97.33%.

### Intrapartum antibiotic usage

Four studies assessed intrapartum antibiotic usage and childhood eczema (Table 2). For better comparability, we used the OR data derived from population data (Table 2). Two of these studies investigated two cohorts, vaginal birth and caesarean section separately^65,66^. Dhudasia et al. found significant risk for both the vaginal birth (OR: 1.28; 95% CI: 1.09; 1.51) and C-section cohorts (OR: 1.48; 95% CI: 1.14; 1.94)^66^. However, Hong et al. only found significant risk in the vaginal birth group (OR: 6.72; 95% CI: 4.73; 9.54) and the C-section cohort produced nonsignificant results (OR: 0.56; 95% CI: 0.28; 1.12)^65^. Overall, the meta-analysis of the included four studies found no statistically significant association with developing atopic dermatitis later in life based on results derived from population data (OR: 1.64; 95% CI: 0.84; 3.17) (Supplementary Figure 6).

### Subgroup analysis

In our meta-analysis, we conducted multiple subgroup analyses examining the following factors: location of the study, type of the study, sample size (number of children involved), mode of the diagnosis of the eczema/atopic dermatitis, age of the children, and the frequency of antibiotic prescription, in order to address potential confounders. Other factors, such as the timing of antibiotic use and the specific antibiotic class, may also influence the measured outcomes; however, there were insufficient data in these domains to statistically assess their potential effects. To analyse the data, we used random effects model, and variables (i.e. OR, aOR) were handled separately. The results of these analyses are summarized in Table 3. Forest plots of the subgroup analyses are found in the supplementary material (Supplementary Figure 7–19).

**Table 3:** Results of the subgroup analyses (significant results are in bold).

**Variable**	**Groups**	**Results**
Location	Europe	OR: 1.25; 95% CI: 1.12; 1.40 (based on 13 studies)^37,39,40,45–47,49,50,52,54,55,60,62^ aOR: 1.40; 95% CI: 1.08; 1.83 (based on 7 studies)^37,39,40,45,46,52,56^
	Asia	OR: 1.27; 95% CI: 1.00; 1.61 (based on 6 studies)^41,44,48,51,59,61^ aOR: 1.47; 95% CI: 0.95; 2.29 (based on 5 studies)^41,44,58,59,61^
	USA	OR: 1.01; 95% CI: 0.65; 1.55 (based on 3 studies)^38,42,43^
Study type	Prospective cohort	OR: 1.11 95% CI 1.05; 1.17 (based on 15 studies)^37–41,44,46,49,51,52,54,55,59,60,62^ aOR: 1.08; 95% CI: 0.99; 1.19 (based on 10 studies)^37,39–41,44,46,52,53,56,59^
	Retrospective cohort	OR: 1.11; 95% CI: 0.81; 1.54 (based on 3 studies)^42,43,48^
Sample size	<1000	OR: 1.52; 95% CI: 1.03; 2.24 (based on 8 studies)^37,38,40,44,45,52,54,62^ aOR: 2.00; 95% CI 1.57; 2.55 (based on 6 studies)^37,40,44,45,52,58^
	1000–10,000	OR: 1.40; 95% CI: 1.24; 1.59 (based on 4 studies)^39,42,46,47^ aOR: 1.11; 95% CI: 0.97; 1.26 (based on 5 studies)^39,42,46,53,56^
	>10,000	OR: 1.14; 95% CI: 1.00; 1.30 (based on 10 studies)^41,43,48–51,55,59–61^ aOR: 1.20; 95% CI: 0.87; 1.66 (based on 3 studies)^41,59,61^
Diagnosis of atopic dermatitis	Medical records	OR: 1.15; 95% CI: 0.95; 1.38 (based on 7 studies)^43,48–51,60,61^
	Parent reported	OR 1.30; 95% CI: 1.13; 1.51 (based on 14 studies)^37–42,44–47,52,54,55,59^ aOR 1.23; 95% CI: 1.06; 1.44 (based on 12 studies)^37,39–42,44–46,52,53,56,59^
Age of child	<18 months	OR: 1.09; 95% CI: 0.96; 1.24 (based on 11 studies)^37,38,40,41,43,44,46,48,54,55,60^ aOR: 1.41; 95% CI: 0.99; 2.01 (based on 7 studies)^37,40,41,44,46,56,58^
	18–60 months	OR: 1.34; 95% CI: 1.00; 1.79 (based on 6 studies)^45,51,52,59,61,62^ aOR: 1.39; 95% CI: 1.05; 1.84 (based on 5 studies)^45,52,53,59,61^
	More than 5 years old	OR 1.30; 95% CI: 1.16; 1.45 (based on 5 studies)^39,42,47,49,50^
The frequency of antibiotic use	Once only	aHR: 1.07; 95% CI: 1.04; 1.11 (based on 3 studies)^49,51,57^
	Twice	aHR: 1.10; 95% CI: 1.05; 1.15 (based on 3 studies)^49,51,57^
	More than two times	aHR: 1.16; 95% CI: 1.10; 1.22 (based on 3 studies)^49,51,57^

Our subgroup analyses revealed differences depending on the geographical location of the studies. The results of the European studies showed a statistically significant association between antibiotic use and atopic dermatitis or eczema, nonetheless, we found non-significant results when we analysed the Asian and American studies. However, it should be noted that only three American studies were involved in this analysis; therefore, these results should be interpreted with caution. Furthermore, there were differences between the results of the prospective and retrospective studies as well. Regarding the OR group, the overall risk was only significant among the prospective studies, but when aOR data was considered, this result became non-significant. It is also noteworthy, that the overall results were non-significant if the diagnosis was made by medical records, but significant when parent reports were used. When compared with the overall effect (OR: 1.24; 95% CI: 1.10–1.38), it is likely that our results were driven by parent-reported studies. However, it is also worth mentioning that six studies in this group reported non-significant results^37,38,41,46,47,59^. When the number of children involved was considered, smaller studies (i.e. those involving fewer than 10,000 children) showed a significant correlation between prenatal antibiotic use and atopic dermatitis. However, when the results of the larger studies were analysed separately, this correlation became non-significant. Regarding the age of the involved children, our study found that the effect of prenatal antibiotic use became significant among children older than 18 months. As for the frequency of antibiotic prescription, our study found that even a single antibiotic course was associated with higher odds of developing atopic dermatitis, and this risk increased with further courses. However, this evidence is also limited due to the low number of studies involved.

### Risk of bias

Two authors independently assessed the risk of bias within the selected studies, and any disagreements were resolved with the help of a third investigator. The results of the risk of bias assessment were discussed when evaluating the limitations of each study. Of the included papers, 25 were judged to have a moderate risk of bias, while six showed a serious risk of bias in at least one domain. Therefore, these studies were considered to have an overall serious risk of bias. However, as shown by the leave-one-out analysis, the results remained significant even after excluding studies with a high risk of bias. (Supplementary Figure 1). Subgroup analyses suggested that part of this heterogeneity may be explained by differences in study characteristics, as significant associations were primarily observed in European studies and prospective cohorts, as well as in studies with smaller sample sizes and parent-reported outcomes, whereas results were attenuated or non-significant in other subgroups.

Studies were judged to be at low risk of bias due to confounding factors if they adjusted for all or almost all major covariates (e.g. breastfeeding, postnatal antibiotic use, maternal allergies, and mode of delivery). Studies that adjusted for fewer factors were considered to be at moderate risk of bias. Studies that made no adjustments were considered to be at serious risk of bias. Bias due to participant selection was low in most cases. However, it was deemed serious in the case of Choi et al. due to their extensive search for controls^43^. Furthermore, studies were judged to be at moderate risk if they only selected mothers with a prior history of allergic diseases. As for the bias in the classification of results studies were mainly judged to be of low risk, but were judged as moderate if no clear definition was given. Deviations from the intended interventions due to missing results were judged to be of low risk of bias for all studies. Studies were judged to be of low risk if they had no missing data. If the number of participants fell by less than 10% compared to the start of the study and the evidence remained robust, the risk of bias was judged to be moderate. If these criteria were not met, the study was judged to be at serious risk of bias. All studies were judged to be at moderate risk of bias in the measurement of outcomes. Regarding the risk of bias in the selection of reported results, all studies were judged to be low risk, as there was no evidence of selective reporting.

After discussion, none of the studies were excluded from the meta-analysis due to risk of bias. (Supplementary Figures 20–21, Supplementary Table 4)

### Grade of evidence

The GRADE assessment revealed that the certainty of the evidence regarding the use of antibiotics during pregnancy and the subsequent development of atopic dermatitis in childhood in these settings was low due to the risk of bias, inconsistency, and imprecision (see Table 4). The certainty of the evidence regarding intrapartum antibiotic use and childhood atopic dermatitis was deemed to be very low due to the small number of included studies, as well as due to risk of bias, inconsistency and imprecision (Table 4).

**Table 4:** The Grade assessment of the included prenatal and intrapartum studies.

Certainty assessment							Number of children		Effect		Certainty		Importance
Number of studies	Study design	Risk of Bias	Inconsistency	Indirectness	Imprecision	Other consi-derations	Antibiotic: yes	Antibiotic: no	Relative (95% CI)	Absolute (95% CI)			
Outcome: Prenatal antibiotic use and prevalence of eczema/atopic dermatitis in children													
26	Non-randomised studies	Serious	Serious	Not serious	Serious	None	1,407,467	2,883,056	-	OR data: 1.24 95% CI (1.10; 1.38) aOR data: 1.32 95% CI (1.12; 1.56) aHR data: 1.10 95% CI (1.05; 1.14)		⨁⨁◯◯ Low	Critical
Outcome: Intrapartum antibiotic use and prevalence of eczema/atopic dermatitis in children													
4	Non-randomised studies	Serious	Serious	Not serious	Serious	None	7,532	10,335	-	OR data: 1.64 95% CI (0.84; 3.17)		⨁◯◯◯ Very low	Critical

## Discussion

This systematic review and meta-analysis aimed to investigate the association between prenatal or intrapartum antibiotic exposure and childhood atopic dermatitis, and provides up-to-date evidence on this relationship. Although several previous studies have examined this association, no prior meta-analysis has undertaken such a comprehensive evaluation of the topic.

Our study identified a statistically significant association between prenatal antibiotic use and childhood AD (OR: 1.24; 95% CI: 1.10; 1.38), which is coherent with several previous studies^21,25–28^ but contrasts with the studies conducted by Tsakok et al. and Baron et al.^22,23^.

Our results remained significant after adjusting publication bias by the trim-and-fill method (OR: 1.21; 95% CI: 1.08; 1.36). The analysis therefore suggested potential left-sided asymmetry, with the adjusted effect size decreasing from OR 1.32 to 1.22, indicating possible overestimation of the observed effect.

The observed effect size (OR: 1.24) indicates a modest but statistically significant increase in the risk of childhood eczema associated with prenatal antibiotic exposure. While the magnitude of the association may appear relatively small at the individual level, it may still be clinically relevant at the population level given the high prevalence of both antibiotic use during pregnancy and atopic dermatitis in childhood. However, it is important to emphasize that the observed association does not imply causality, and the results should be interpreted with caution due to the observational nature of the included studies and the potential for residual confounding. Even though beta-lactams, fosfomycin and clindamycin are considered to be safe during pregnancy^67^ our results advise caution regarding antibiotic use during this period. In pregnancy, antimicrobial therapy should be used judiciously—only when clearly indicated, for the shortest effective duration, and with the narrowest-spectrum agents possible, preferably guided by microbiological confirmation of the causative pathogen. Although substantial heterogeneity was observed across studies, this variability is likely attributable to differences in study design, population characteristics, outcome definitions, and exposure assessment. However, this still contrasts with the study of Huang et al. who identified zero heterogeneity (I^2^ = 0.0%, P = 0.765), but this is likely due to the fact that they only investigated eczema before 1 year of age^25^. Importantly, the direction of effect remained consistent across sensitivity and subgroup analyses. A recent meta-analysis, based on 16 prenatal and 4 intrapartum studies, with an overall number of children of 3,256,929, also investigated the associations between prenatal antibiotic use and childhood atopic dermatitis. The present analysis includes a substantially larger number of studies than previous meta-analyses on this topic. With the exception of Wan et al., which included 18 studies^28^, earlier analyses incorporated only 3 to 8 relevant studies^21–27^. Similarly to our study, Chang et al. found significant risk with an OR of 1.12 (95% CI: 1.03; 1.31), but their results became non-significant after adjusting publication bias^29^. When hazard ratio (HR) data were examined, the reported associations were non-significant. This contrasts with our study, since we found a consistent association even when adjusted hazard ratios were considered. On the other hand, when intrapartum antibiotic use was considered, our study did not find a significant risk, which is coherent with the results of Chang et al^29^. This might be explained by the much narrower timing of exposure, leading only to a transient impact on the infant’s gut microbiome^29^. However, it is important to note that the duration of intrapartum antibiotic exposure was only investigated by Wohl et al., and they found a significant risk when the exposure was longer than 24 hours^63^. The low number of studies in this category limits our results. It is also worth mentioning that the heterogeneity was also high among the studies (I^2^ 95.6%), Further analysis of this issue is therefore needed. One major strength of our study is the high number of included subjects. This is due to the large initial ‘study pool’, and extensive search for relevant studies. However, it is important to note that grey literature (e.g. PhD theses) was excluded from our meta-analysis. Another major strength of our study is transparency. Our study was registered with PROSPERO, and we predefined the research question, the population, the comparator and the outcome. We carried out the systematic review and meta-analysis as outlined in the original protocol, making no alterations to the objectives, eligibility criteria, search strategy or methods. This resulted in several studies being included that had not been assessed prior to the analysis. Excluding grey literature also resulted in a distinct pool of included studies.

The large number of included studies enabled us to perform various subgroup analyses. This is in contrast with other meta analyses that analysed eczema as an outcome, but did not make further distinctions^23–25^. As the results of the original studies were presented using different parameters, we did not convert them to avoid potential inaccuracy. Therefore, the results of these studies were analysed separately. Some studies provided multiple parameters and were included in multiple groups.

Comparing our subgroup analyses three meta-analyses^21,28,29^ also separated studies based on sample size, four studies addressed the different study types^21,22,28,29^. Wan et al. also addressed the study location^28^ and Chang et al. separated studies based on number of courses administered similarly to our study^29^. In contrast with our subgroup analysis, two meta analyses differentiated studies based on publication year^21,29^, and also timing of exposure^28,29^. However, in the case of Chang et al. only two studies were included^29^, and Wan et al. separated them as follows: antibiotic use before delivery, all trimesters and 3^rd^ trimester specifically^28^. Interestingly, none of the included meta-analyses made a subgroup analysis based on the age of the children at the time of the diagnosis.

Another strength of our study is that we assessed not only the risk of bias in the initial studies, but also publication bias, and performed a leave-one-out analysis. We also investigated the intrapartum studies separately due to the specific timing of antibiotic use. As these studies provided results using various parameters, we used population-data-derived ORs to improve comparability. By analysing intrapartum and prenatal exposures separately—reflecting their substantially different timing and duration—the present study addresses a methodological limitation of some previous meta-analyses, in which this distinction was not made, potentially leading to bias^23,24,28^.

However, our meta-analysis is not without limitations. A substantial proportion of the included studies relied on parent-reported diagnoses of atopic dermatitis, which may be subject to recall bias or misclassification. Although subgroup analyses stratified by diagnostic method yielded broadly consistent results, this limitation should be considered when interpreting the magnitude of the observed associations. Given the observational design of the included studies, all findings should be interpreted as associations rather than evidence of a causal relationship. One further limitation is the low grade of evidence, based on the GRADE analysis, due to the high heterogeneity, wide confidence intervals and an overall serious risk of bias. Another major limitation is the limited aOR data regarding various subgroups, leading to potential bias between the results. Furthermore, among the studies that did adjust to various confounders, not all calculated for postnatal antibiotic use, which can affect the results. Therefore, residual confounding cannot be excluded, particularly with respect to postnatal antibiotic exposure, which was not consistently adjusted for across the included studies. Given the known impact of early-life antibiotic use on immune development, this factor may have influenced the reported associations independently of prenatal exposure. Studies based on medical reports may not have had access to the children’s postnatal antibiotic use data.

Furthermore, there is limited data on the duration, dosage and type of antibiotic used, as well as the mode and timing of administration. While some studies addressed these matters, there was insufficient data to enable comparisons to be made. In the case of the timing of antibiotic use, the control group and parameters varied between studies; therefore, no statistical analysis could be made. Six studies provided relevant data in total. Among them, in three cases the control group was ‘no exposure in the given trimester’^49,56,59^, while in two other cases the reference group was no antibiotic exposure during pregnancy, and only study gave data in both categories^47,48,51^. One Further assessments need to be carried out regarding this issue. Additionally, the majority of the included studies used parent reported data, and even though these studies used specific questions for eczema assessment this is not without potential bias. However, Kurzius Spencer et al. used two questionnaires and one medical report for outcome assessment and they found a significant concordance with 80 and 81%, although the low Kappa values represent only marginal reproducibility^38^.

## Conclusion

Overall, our meta-analysis demonstrated a consistent and statistically significant association between prenatal antibiotic exposure and an increased risk of atopic dermatitis and eczema in offspring. These findings underscore the importance of heightened awareness regarding antibiotic use during pregnancy, particularly in light of potential long-term immunological consequences for the child. While antibiotics remain essential in managing maternal infections, our results support the need for careful risk–benefit consideration and more judicious prescribing practices during the prenatal period. The association was not confirmed when studies assessing the use intrapartum antibiotics were examined. It should also be noted that limited data is available on the type of antibiotics used, their exact dosage and the timing of exposure. Further studies may provide the additional detail needed to better characterize these associations.

## Supplementary Information

Supplementary Information.

## Data Availability

Data extracted from the included studies that is not reported in the article or in the supporting information can be obtained from the authors.

## Abbreviations

95% CI: 95% Confidence interval
AD: Atopic dermatitis
aHR: Adjusted hazard ratio
aOR: Adjusted odds ratio
GRADE: Grading of recommendations assessment, development and evaluation
HR: Hazard ratio
OR: Odds ratio
RR: Relative risk
PICO: Patients, intervention, comparison, outcome, format
