# Supplementary material for: Prenatal maternal antibiotic use increases the risk of childhood eczema: a systematic review and meta-analysis
Source: Sci Rep. 2026 May 8;16:21156. doi: 10.1038/s41598-026-51441-x (PMC13342322; doi:10.1038/s41598-026-51441-x)
Supplement: Supplementary file 1 — Supplementary Information. [file 41598_2026_51441_MOESM1_ESM.docx]

**Prenatal maternal antibiotic use increases the risk of childhood eczema: a systematic review and meta-analysis**

Szilárd Petrás^a^, Bettina Vargáné Szabó^a^, Tivadar Kiss^b^, Muh. Akbar Bahar^c^, Dezső Csupor^a,d^, Barbara Tóth^a^

**Affiliations**

^a^ Institute of Clinical Pharmacy, Faculty of Pharmacy, University of Szeged, Szeged, Hungary

^b^ Institute of Pharmacognosy, Faculty of Pharmacy, University of Szeged, Szeged, Hungary

^c^ Department of Pharmacy, Faculty of Pharmacy, Universitas Hasanuddin, Makassar, Indonesia

^d^ Institute of Translational Medicine, University of Pécs, Pécs, Hungary

Correspondence:

Barbara Tóth

Institute of Clinical Pharmacy, Faculty of Pharmacy, University of Szeged, Szeged, Hungary

H-6725 Szeged, Szikra u. 6.

Phone/Fax: +36-62-544-921

toth.barbara.eva@szte.hu

**Supplementary Table 1**. List of excluded studies and exclusion reason

| **Primary exclusion reason** | **Reference number** |
| --- | --- |
| Not corresponding to our search | [1] – [103] |
| Not an independent publication | [104] – [118] |
| No full text available | [119] – [127] |

1. Yazar B, Meydanlioglu A. The prevalence and associated factors of asthma, allergic rhinitis, and eczema in Turkish children and adolescents. *Pediatr Pulmonol*. 2022;57(10):2491-2501. doi:10.1002/ppul.26065
2. Ahmadizar F, Vijverberg SJH, Arets HGM, et al. Early-life antibiotic exposure increases the risk of developing allergic symptoms later in life: A meta-analysis. Allergy. 2018;73(5):971-986. doi:10.1111/all.13332
3. Ahmadizar F, Vijverberg SJH, Arets HGM, et al. Early life antibiotic exposure is associated with an increased risk of allergy. *Eur Respir J*. 2016;48((Ahmadizar F.; Vijverberg S.J.H.; Arets H.G.M.; De Boer A.; Lang J.E.; Garssen J.; Kraneveld A.; Maitland-van der Zee A.H.)). doi:10.1183/13993003.congress-2016.PA3639
4. Anderson SE, Weatherly L, Shane HL. Contribution of antimicrobials to the development of allergic disease. *Curr Opin Immunol*. 2019;60:91-95. doi:10.1016/j.coi.2019.05.003
5. Arrais M, Lulua O, Quifica F, Rosado-Pinto J, Gama JMR, Taborda-Barata L. Prevalence of asthma, allergic rhinitis and eczema in 6-7-year-old schoolchildren from Luanda, Angola. *Allergol Immunopathol (Madr)*. 2019;47(6):523-534. doi:10.1016/j.aller.2018.12.002
6. Ting JY, Roberts A. Association of early life antibiotics and health outcomes: Evidence from clinical studies. *Semin Perinatol*. 2020;44(8):151322. doi:10.1016/j.semperi.2020.151322
7. Bookstaver PB, Bland CM, Griffin B, Stover KR, Eiland LS, McLaughlin M. A Review of Antibiotic Use in Pregnancy. *Pharmacotherapy*. 2015;35(11):1052-1062. doi:10.1002/phar.1649
8. Cantarutti A, Amidei CB, Bonaugurio AS, Rescigno P, Canova C. Early-life exposure to antibiotics and subsequent development of atopic dermatitis. *Expert Rev Clin Pharmacol*. 2022;15(6):779-785. doi:10.1080/17512433.2022.2092471
9. Carstens LE, Westerbeek EA, van Zwol A, van Elburg RM. Neonatal antibiotics in preterm infants and allergic disorders later in life. *Pediatr Allergy Immunol*. 2016;27(7):759-764. doi:10.1111/pai.12614
10. Celedón JC, Litonjua AA, Ryan L, Weiss ST, Gold DR. Lack of association between antibiotic use in the first year of life and asthma, allergic rhinitis, or eczema at age 5 years. *Am J Respir Crit Care Med*. 2002;166(1):72-75. doi:10.1164/rccm.2109074
11. Celedón JC, Weiss ST. Use of antibacterials in infancy: clinical implications for childhood asthma and allergies. *Treat Respir Med*. 2004;3(5):291-294. doi:10.2165/00151829-200403050-00003
12. Chawla KK, Nowak-Wegrzyn A. Antibiotic use in the first year of life and risk of atopic disease in early childhood. *Pediatrics*. 2009;124(SUPPL. 2):S107-S108. doi:10.1542/peds.2009-1870E.
13. Chinratanapisit S, Suratannon N, Pacharn P, Sritipsukho P, Vichyanond P. Prevalence and risk factors of allergic rhinitis in children in Bangkok area. *Asian Pac J Allergy Immunol*. 2019;37(4):232-239. doi:10.12932/AP-120618-0337
14. Choi CW, Yang BR, Suh DI, et al. Infection, antibiotic exposure and development of atopic dermatitis: A nationwide case-control study. *J Dermatol*. 2020;47(7):707-713. doi:10.1111/1346-8138.15387
15. Choi E, Song KB, Park MJ, et al. Effect of antibiotic use during early life on the persistence and severity of allergic rhinitis in school-aged children: COCOA study. *Allergy Eur J Allergy Clin Immunol*. 2023;78((Choi E.; Song K.B.; Lee S.Y.; Hong S.J.) Asan Medical Center, Seoul, South Korea):579. doi:10.1111/all.15616
16. Cohet C, Cheng S, MacDonald C, et al. Infections, medication use, and the prevalence of symptoms of asthma, rhinitis, and eczema in childhood. *J Epidemiol Community Health*. 2004;58(10):852-857. doi:10.1136/jech.2003.019182
17. Crane J. Pro and anti: the biotics of allergic disease. *Thorax*. 2002;57 Suppl 2(Suppl 2):II40-II46.
18. Deng J, Parthasarathy V, Bordeaux Z, et al. Risk factors and temporal associations of progression of the atopic march in children with atopic dermatitis. *Br J Dermatol*. 2022;187(3):e114. doi:10.1111/bjd.21666
19. Dietert RR, Zelikoff JT. Early-life environment, developmental immunotoxicology, and the risk of pediatric allergic disease including asthma. Birth Defects Res B Dev Reprod Toxicol. 2008 Dec;83(6):547-60. doi: 10.1002/bdrb.20170. PMID: 19085948.
20. Droste JH, Wieringa MH, Weyler JJ, Nelen VJ, Vermeire PA, Van Bever HP. Does the use of antibiotics in early childhood increase the risk of asthma and allergic disease?. *Clin Exp Allergy*. 2000;30(11):1547-1553. doi:10.1046/j.1365-2222.2000.00939.x
21. Duong QA, Pittet LF, Curtis N, Zimmermann P. Antibiotic exposure and adverse long-term health outcomes in children: A systematic review and meta-analysis. *J Infect*. 2022;85(3):213-300. doi:10.1016/j.jinf.2022.01.005
22. Eigenmann P. Antibiotic use favors early-life allergies, intrauterine blood flow may influence respiratory allergies, and features of hyper-IgE syndrome. *Pediatr Allergy Immunol*. 2019;30(4):403-404. doi:10.1111/pai.13062
23. Feng H, Xiong X, Chen Z, et al. Prevalence and Influencing Factors of Food Allergy in Global Context: A Meta-Analysis. *Int Arch Allergy Immunol*. 2023;184(4):320-352. doi:10.1159/000527870**.**
24. Fernández SV, Urbina J, Castello MA, et al. Prevalence, incidence and associated risk factors of adverse reaction to food in Cuban infants - a population-based prospective study. *World Allergy Organ J*. 2016;9((Fernández S.V.; Medina R.S.; Hernández H.F.) National Institute of Hygiene Epidemiology and Microbiology, Cuba). doi:10.1186/s40413-016-0097-0
25. Gao Y, Nanan R, Macia L, et al. The maternal gut microbiome during pregnancy and offspring allergy and asthma. *J Allergy Clin Immunol*. 2021;148(3):669-678. doi:10.1016/j.jaci.2021.07.011
26. Garcia-Marcos L, González-Díaz C, Garvajal-Urueña I, et al. Early exposure to paracetamol or to antibiotics and eczema at school age: modification by asthma and rhinoconjunctivitis. *Pediatr Allergy Immunol*. 2010;21(7):1036-1042. doi:10.1111/j.1399-3038.2010.01037.x
27. Ho CL, Chang LI, Wu WF. The prevalence and risk factors of atopic dermatitis in 6-8 year-old first graders in Taipei. *Pediatr Neonatol*. 2019;60(2):166-171. doi:10.1016/j.pedneo.2018.05.010
28. Ho CL, Wu WF. Risk factor analysis of allergic rhinitis in 6-8 year-old children in Taipei. *PLoS One*. 2021;16(4):e0249572. Published 2021 Apr 2. doi:10.1371/journal.pone.0249572
29. Hoskin-Parr L, Teyhan A, Blocker A, Henderson AJ. Antibiotic exposure in the first two years of life and development of asthma and other allergic diseases by 7.5 yr: a dose-dependent relationship. *Pediatr Allergy Immunol*. 2013;24(8):762-771. doi:10.1111/pai.12153
30. Hoskinson C, Medeleanu MV, Reyna ME, et al. Antibiotics taken within the first year of life are linked to infant gut microbiome disruption and elevated atopic dermatitis risk. *J Allergy Clin Immunol*. 2024;154(1):131-142. doi:10.1016/j.jaci.2024.03.025
31. Jones K, Sitarik A, Havstad S, et al. Association between antibiotic treatment in the first six months of life and clinical allergic outcomes at ages 2 to 3 years. *J Allergy Clin Immunol*. 2014;133(2):AB235. doi:10.1016/j.jaci.2013.12.837
32. Kamphorst K, Vlieger AM, Oosterloo BC, Waarlo S, van Elburg RM. Higher risk of allergies at 4-6 years of age after systemic antibiotics in the first week of life. *Allergy*. 2021;76(8):2599-2602. doi:10.1111/all.14829
33. Karimi M, Mirzaei M. Antibiotic use and symptoms of asthma, allergic rhinitis and eczema in children. *Iran J Pediatr*. 2009;19(2):141-146
34. Dowhower Karpa K, Paul IM, Leckie JA, et al. A retrospective chart review to identify perinatal factors associated with food allergies. *Nutr J*. 2012;11:87. Published 2012 Oct 19. doi:10.1186/1475-2891-11-87
35. Kim SW. The effect of antibiotics in children and adolescent developing asthma and allergic diseases. *J Allergy Clin Immunol*. 2018;141(2):AB8.
36. Kocijancic LB. Maternal antibiotic use during lactation and the development of asthma and atopic dermatitis in Slovenian children. *ALLERGY*. 2007;62(83):405.
37. Koplin JJ, Martin PE, Tang MLK, et al. Do factors known to alter infant microbial exposures alter the risk of food allergy and eczema in a population-based infant study? *J Allergy Clin Immunol*. 2012;129(2):AB231. doi:10.1016/j.jaci.2011.12.150
38. Kummeling I, Thijs C, Penders J. Early life antibiotic use and risk of allergy and asthma - A systematic review and meta-analysis of reverse causation and confounding-by-indication. *Allergy Eur J Allergy Clin Immunol*. 2010;65((Kummeling I.; Thijs C.; Penders J.) Maastricht University, Epidemiology, Maastricht, Netherlands):189-190. doi:10.1111/j.1398-9995.2010.02392.x
39. Kummeling I, Stelma FF, Dagnelie PC, et al. Early life exposure to antibiotics and the subsequent development of eczema, wheeze, and allergic sensitization in the first 2 years of life: the KOALA Birth Cohort Study. *Pediatrics*. 2007;119(1):e225-e231. doi:10.1542/peds.2006-0896
40. Kusel MM, de Klerk N, Holt PG, Sly PD. Antibiotic use in the first year of life and risk of atopic disease in early childhood. *Clin Exp Allergy*. 2008;38(12):1921-1928. doi:10.1111/j.1365-2222.2008.03138.x
41. Lishman H, Nickel NC, Sbihi H, et al. Investigating the effect of early life antibiotic use on asthma and allergy risk in over 600 000 Canadian children: a protocol for a retrospective cohort study in British Columbia and Manitoba. *BMJ Open*. 2023;13(4):e067271. doi:10.1136/bmjopen-2022-067271
42. Liu X, Wu R, Fu Y, et al. Meta-analysis of early-life antibiotic use and allergic rhinitis. *Open Med (Wars)*. 2022;17(1):1760-1772. Published 2022 Nov 4. doi:10.1515/med-2022-0459
43. Wang JY, Liu LF, Chen CY, Huang YW, Hsiung CA, Tsai HJ. Acetaminophen and/or antibiotic use in early life and the development of childhood allergic diseases. *Int J Epidemiol*. 2013;42(4):1087-1099. doi:10.1093/ije/dyt121
44. Love BL, Mann JR, Hardin JW, Lu ZK, Cox C, Amrol DJ. Antibiotic prescription and food allergy in young children. *Allergy Asthma Clin Immunol*. 2016;12:41. Published 2016 Aug 17. doi:10.1186/s13223-016-0148-7
45. Mai XM, Kull I, Wickman M, Bergström A. Antibiotic use in early life and development of allergic diseases: respiratory infection as the explanation. *Clin Exp Allergy*. 2010;40(8):1230-1237. doi:10.1111/j.1365-2222.2010.03532.x
46. Marrs T, Bruce KD, Logan K, et al. Is there an association between microbial exposure and food allergy? A systematic review. *Pediatr Allergy Immunol*. 2013;24(4):311-320.e8. doi:10.1111/pai.12064.
47. Matson MD, Enweasor C, Tran L, Odudu G. 18-MONTH-OLD PRESENTING WITH CELLULITIS AND ATOPIC DERMATITIS REFRACTORY TO ANTIBIOTIC AND STEROID TREATMENT. *ANNALS OF ALLERGY ASTHMA & IMMUNOLOGY*. 2023;131(5, S):S138.
48. McKeever TM, Lewis SA, Smith C, et al. Early exposure to infections and antibiotics and the incidence of allergic disease: a birth cohort study with the West Midlands General Practice Research Database. *J Allergy Clin Immunol*. 2002;109(1):43-50. doi:10.1067/mai.2002.121016
49. ae E, Susi A, Kropp LE, Schwartz DJ, Gorman GH, Nylund CM. Association Between Use of Acid-Suppressive Medications and Antibiotics During Infancy and Allergic Diseases in Early Childhood. *JAMA Pediatr*. 2018;172(6):e180315. doi:10.1001/jamapediatrics.2018.0315
50. Moriarty B, Powell AM, Yeo L, Flohr C. Does early life exposure to antibiotics increase the risk of atopic eczema? A systematic review. *Br J Dermatol*. 2011;165((Moriarty B.; Powell A.M.; Yeo L.; Flohr C.) Department of Paediatric Dermatology, St John’s Institute of Dermatology, Guy’s and St Thomas’ Hospitals NHS Foundation Trust, London, United Kingdom):116. doi:10.1111/j.1365-2133.2011.10289.x
51. Munivrana Skvorc H, Plavec D, Munivrana S, Skvorc M, Nogalo B, Turkalj M. Antibiotic and antipyretic use in first years of life and development of symptoms of allergy diseases in Croatian children. *Allergy Eur J Allergy Clin Immunol*. 2011;66((Munivrana Skvorc H.; Plavec D.; Turkalj M.) Children’s Hospital Srebrnjak, Department of allergy and pulmology, Zagreb, Croatia):553-554. doi:10.1111/j.1398-9995.2011.02608.x
52. Munyaka PM, Khafipour E, Ghia JE. External influence of early childhood establishment of gut microbiota and subsequent health implications. *Front Pediatr*. 2014;2:109. Published 2014 Oct 9. doi:10.3389/fped.2014.00109
53. Narla S, Silverberg JI. The Role of Environmental Exposures in Atopic Dermatitis. *Curr Allergy Asthma Rep*. 2020;20(12):74. Published 2020 Oct 12. doi:10.1007/s11882-020-00971-z
54. Nguyen AT, Aquino M. Atopic Dermatitis and Eczema: Association Between Early Life Antibiotic Exposure and Development of Early Childhood Atopic Dermatitis. *Pediatrics*. 2023;152((Nguyen A.T.; Aquino M.) Providence, RI, United States):S21-S22. doi:10.1542/peds.2023-064344H
55. Ohya Y, Yonemoto J, Ogata T, et al. Influence of environmental chemicals and drugs taken before and during pregnancy on onset of childhood asthma and eczema. *Allergy Eur J Allergy Clin Immunol*. 2011;66((Ohya Y.; Ogata T.; Sakamoto N.; Qiu D.; Yamamoto K.; Shoda T.; Futamura M.; Narita M.; Tsumura Y.; Hamaguchi M.; Doi M.; Horikawa R.; Aizawa S.; Kitagawa M.; Saito H.) National Center for Child Health and Development, Tokyo, Japan):554. doi:10.1111/j.1398-9995.2011.02608.x
56. Oosterloo BC, Rutten NB, Van Elburg RM, et al. Neonatal antibiotic treatment is associated with an increased risk for wheezing and allergic sensitization in the first year of life. *Allergy Eur J Allergy Clin Immunol*. 2017;72((Oosterloo B.C.) Emma Children’s Hospital, AMC, Amsterdam, Netherlands):364. doi:10.1111/all.13248
57. Pang K, Li G, Li M, et al. Prevalence and Risk Factors for Allergic Rhinitis in China: A Systematic Review and Meta-Analysis. *Evid Based Complement Alternat Med*. 2022;2022:7165627. Published 2022 Sep 23. doi:10.1155/2022/7165627**.**
58. Park YM, Lee SY, Kim WK, et al. Risk factors of atopic dermatitis in Korean schoolchildren: 2010 international study of asthma and allergies in childhood. *Asian Pac J Allergy Immunol*. 2016;34(1):65-72. doi:10.12932/AP0621.34.1.2016
59. Peldan P, Kukkonen AK, Savilahti E, Kuitunen M. Perinatal probiotics decreased eczema up to 10 years of age, but at 5-10 years, allergic rhino-conjunctivitis was increased. *Clin Exp Allergy*. 2017;47(7):975-979. doi:10.1111/cea.12924
60. Prince BT, Mandel MJ, Nadeau K, Singh AM. Gut Microbiome and the Development of Food Allergy and Allergic Disease. *Pediatr Clin North Am*. 2015;62(6):1479-1492. doi:10.1016/j.pcl.2015.07.007
61. Raciborski F, Tomaszewska A, Komorowski J, et al. The relationship between antibiotic therapy in early childhood and the symptoms of allergy in children aged 6-8 years - the questionnaire study results. *Int J Occup Med Environ Health*. 2012;25(4):470-480. doi:10.2478/S13382-012-0056-0
62. Sandini U, Kukkonen AK, Poussa T, Sandini L, Savilahti E, Kuitunen M. Protective and risk factors for allergic diseases in high-risk children at the ages of two and five years. *Int Arch Allergy Immunol*. 2011;156(3):339-348. doi:10.1159/000323907
63. Sarkar A, Yoo JY, Valeria Ozorio Dutra S, Morgan KH, Groer M. The Association between Early-Life Gut Microbiota and Long-Term Health and Diseases. *J Clin Med*. 2021;10(3):459. Published 2021 Jan 25. doi:10.3390/jcm10030459
64. Schoch JJ, Satcher KG, Garvan CW, Monir RL, Neu J, Lemas DJ. Association between early life antibiotic exposure and development of early childhood atopic dermatitis. *JAAD Int*. 2022;10:68-74. Published 2022 Nov 13. doi:10.1016/j.jdin.2022.11.002
65. Schmitt I, Schmitt NM, Kirch W, Meurer M. Antibiotic exposure in infancy is a risk factor for incident atopic eczema in the second year of life: A population-based cohort study. *JOURNAL OF INVESTIGATIVE DERMATOLOGY*. 2008;128(1):S84.
66. Schmitt J, Schmitt NM, Kirch W, Meurer M. Early exposure to antibiotics and infections and the incidence of atopic eczema: a population-based cohort study. *Pediatr Allergy Immunol*. 2010;21(2 Pt 1):292-300. doi:10.1111/j.1399-3038.2009.00901.x
67. Sestito S, D'Auria E, Baldassarre ME, et al. The Role of Prebiotics and Probiotics in Prevention of Allergic Diseases in Infants. *Front Pediatr*. 2020;8:583946. Published 2020 Dec 22. doi:10.3389/fped.2020.583946
68. Shen M, Xiao Y, Li J, Chen X. 260 Use of antibiotics in preschool age predicts atopic and allergic skin diseases in adolescents: A retrospective cohort study. *J Invest Dermatol*. 2019;139(5):S45. doi:10.1016/j.jid.2019.03.336
69. Scranton SE, Davis KL. The association of early life exposure to antibiotics and the development of asthma, eczema and atopy in a birth cohort: Confounding or causality? *Pediatrics*. 2009;124(SUPPL. 2):S108-S109. doi:10.1542/peds.2009-1870F
70. Slob EMA, Brew BK, Vijverberg SJH, et al. Early-life antibiotic use and risk of asthma and eczema: results of a discordant twin study. *Eur Respir J*. 2020;55(4):1902021. Published 2020 Apr 23. doi:10.1183/13993003.02021-2019
71. Slob EMA, Kats CJAR, Vijverberg SJH, et al. Early-life antibiotics use increases the risk of asthma and eczema: A discordant twin study. *Pharmacoepidemiol Drug Saf*. 2019;28((Slob E.M.A.; Kats C.J.A.R.; Vijverberg S.J.H.; Maitland-Van Der Zee A.H.) Academic Medical Center, Amsterdam Public Health Research Institute, Amsterdam, Netherlands):445. doi:10.1002/pds.4864
72. Sobko T, Schiött J, Ehlin A, Lundberg J, Montgomery S, Norman M. Neonatal sepsis, antibiotic therapy and later risk of asthma and allergy. *Paediatr Perinat Epidemiol*. 2010;24(1):88-92. doi:10.1111/j.1365-3016.2009.01080.x
73. Sultész M, Katona G, Hirschberg A, Gálffy G. Prevalence and risk factors for allergic rhinitis in primary schoolchildren in Budapest. *Int J Pediatr Otorhinolaryngol*. 2010;74(5):503-509. doi:10.1016/j.ijporl.2010.02.008
74. Tamay Z, Akcay A, Ones U, Guler N, Kilic G, Zencir M. Prevalence and risk factors for allergic rhinitis in primary school children. *Int J Pediatr Otorhinolaryngol*. 2007;71(3):463-471. doi:10.1016/j.ijporl.2006.11.013
75. Tong X, Tong H, Gao L, et al. A Multicenter Study of Prevalence and Risk Factors for Allergic Rhinitis in Primary School Children in 5 Cities of Hubei Province, China. *Int Arch Allergy Immunol*. 2022;183(1):34-44. doi:10.1159/000517948
76. Tuniyazi M, Li S, Hu X, Fu Y, Zhang N. The Role of Early Life Microbiota Composition in the Development of Allergic Diseases. *Microorganisms*. 2022;10(6):1190. Published 2022 Jun 9. doi:10.3390/microorganisms10061190
77. Uday Kiran G, Vinod Kumar P, Hareesh R. EARLY EXPOSURE TO ANTIBIOTICS AND THE DEVELOPMENT OF ATOPIC DISEASES: A RETROSPECTIVE COHORT ANALYSIS. *Int J Acad Med Pharm*. 2024;6(3):137-141. doi:10.47009/jamp.2024.6.3.30
78. Uppell K. ANTIBIOTIC USE IN INFANCY LINKED TO CHILDHOOD ECZEMA. *EUROPEAN JOURNAL OF HOSPITAL PHARMACY-SCIENCE AND PRACTICE*. 2013;20(5):321.
79. Vandenplas Y, Veereman-Wauters G, De Greef E, et al. Probiotics and prebiotics in prevention and treatment of diseases in infants and children. *J Pediatr (Rio J)*. 2011;87(4):292-300. doi:10.2223/JPED.2103
80. Verhulst SL, Vael C, Beunckens C, Nelen V, Goossens H, Desager K. A longitudinal analysis on the association between antibiotic use, intestinal microflora, and wheezing during the first year of life. *J Asthma*. 2008;45(9):828-832. doi:10.1080/02770900802339734
81. Wang JY, Liu LF, Chen CY, Huang YW, Hsiung CA, Tsai HJ. Acetaminophen and/or antibiotic use in early life and the development of childhood allergic diseases. *Int J Epidemiol*. 2013;42(4):1087-1099. doi:10.1093/ije/dyt121
82. Wibowo N, Mose JC, Karkata MK, et al. The status of probiotics supplementation during pregnancy. *MEDICAL JOURNAL OF INDONESIA*. 2015;24(2):120-130. doi:10.13181/mji.v24i2.1223
83. Wickens K, Ingham T, Epton M, et al. The association of early life exposure to antibiotics and the development of asthma, eczema and atopy in a birth cohort: confounding or causality?. *Clin Exp Allergy*. 2008;38(8):1318-1324. doi:10.1111/j.1365-2222.2008.03024.x
84. Yamamoto-Hanada K, Yang L, Narita M, Saito H, Ohya Y. Influence of antibiotic use in early childhood on asthma and allergic diseases at age 5. *Ann Allergy Asthma Immunol*. 2017;119(1):54-58. doi:10.1016/j.anai.2017.05.013
85. Yildizdas HY, Oezcan A, Sertdemir Y, Yilmaz M. Effect of healthcare associated infections and broad spectrum antibiotic use in newborn period on development of asthma, allergic rhinitis and atopic dermatitis in early childhood. *CUKUROVA MEDICAL JOURNAL*. 2017;42(1):132-139. doi:10.17826/cutf.280160
86. Yoon J. Risk Factors of Allergic Rhinitis in Preschool Children and Clinical Utility of Feno. *World Allergy Organ J*. 2016;9(SUPPL.1):60. doi:10.1186/s40413-016-0096-1
87. Yudina YV, Korsunskiy AA, Aminova AI, et al. Current concepts of intestinal dysbiosis as a risk factor for atopic dermatitis in children. *Vopr Prakt Pediatr*. 2019;14(4):44-50. doi:10.20953/1817-7646-2019-4-44-50
88. Zou Z, Liu W, Huang C, Sun C, Zhang J. First-Year Antibiotics Exposure in Relation to Childhood Asthma, Allergies, and Airway Illnesses. *Int J Environ Res Public Health*. 2020;17(16):5700. Published 2020 Aug 7. doi:10.3390/ijerph17165700
89. Zven SE, Susi A, Mitre E, Nylund CM. Association Between Use of Multiple Classes of Antibiotic in Infancy and Allergic Disease in Childhood. *JAMA Pediatr*. 2020;174(2):199-200. doi:10.1001/jamapediatrics.2019.4794
90. Brandt S, Thorsen J, Rasmussen MA, et al. Use of antibiotics in early life and development of diseases in childhood: nationwide registry study. *BMJ Med*. 2025;4(1):e001064. Published 2025 Mar 11. doi:10.1136/bmjmed-2024-001064.
91. Mousavian AH, Zare Garizi F, Ghoreshi B, et al. The association of infant and mother gut microbiomes with development of allergic diseases in children: a systematic review. *J Asthma*. 2024;61(10):1121-1135. doi:10.1080/02770903.2024.2332921**.**
92. Räty S, Ollila H, Turta O, et al. Neonatal and early infancy antibiotic exposure is associated with childhood atopic dermatitis, wheeze and asthma. *Eur J Pediatr*. 2024;183(12):5191-5202. doi:10.1007/s00431-024-05775-1.
93. Li W, Li A. Exploring the causal relationship between gut microbiota and atopic dermatitis: A Mendelian randomization study. *Medicine (Baltimore)*. 2024;103(52):e40193. doi:10.1097/MD.0000000000040193.
94. Lu X, Shi Z, Jiang L, Zhang S. Maternal gut microbiota in the health of mothers and offspring: from the perspective of immunology. *Front Immunol*. 2024;15((Lu X.; Jiang L., linglingjiang@zju.edu.cn; Zhang S., zhangsongying@zju.edu.cn) Assisted Reproduction Unit, Department of Obstetrics and Gynecology, Sir Run Run Shaw Hospital, Zhejiang University School of Medicine, Hangzhou, China). doi:10.3389/fimmu.2024.1362784
95. Kim S, Suh DH, Lee S, Kim HS, Cho SH, Woo YR. Associations Between Skin Microbiome and Metabolome in the Pathogenesis of Atopic Dermatitis Patients With Scalp Involvement. *Allergy Asthma Immunol Res*. 2024;16(6):668-681. doi:10.4168/aair.2024.16.6.668
96. Panduru M, Panduru NM, Ion DA. Antibiotherapy in the first year of life and atopic dermatitis. *Ther Pharmacol Clin Toxicol*. 2012;16(3):215-219
97. Dodge L, Gao S, Sinnott S, et al. Characterization of atopic dermatitis medication use before and during pregnancy in the United States. *Br J Dermatol*. 2024;191. doi:10.1093/bjd/ljae266.074
98. Foliaki S, Nielsen SK, Björkstén B, et al. Antibiotic sales and the prevalence of symptoms of asthma, rhinitis, and eczema: The International Study of Asthma and Allergies in Childhood (ISAAC). *Int J Epidemiol*. 2004;33(3):558-563. doi:10.1093/ije/dyh031
99. von Mutius E, Illi S, Hirsch T, Leupold W, Keil U, Weiland SK. Frequency of infections and risk of asthma, atopy and airway hyperresponsiveness in children. *Eur Respir J*. 1999;14(1):4-11. doi:10.1034/j.1399-3003.1999.14a03.x
100. Wen HJ, Chen PC, Chiang TL, Lin SJ, Chuang YL, Guo YL. Predicting risk for early infantile atopic dermatitis by hereditary and environmental factors. *Br J Dermatol*. 2009;161(5):1166-1172. doi:10.1111/j.1365-2133.2009.09412.x
101. Mommers M, Thijs C, Stelma F, et al. Timing of infection and development of wheeze, eczema, and atopic sensitization during the first 2 yr of life: the KOALA Birth Cohort Study. *Pediatr Allergy Immunol*. 2010;21(6):983-989. doi:10.1111/j.1399-3038.2010.01042.x
102. Choi EY, Bea S, Lee H, et al. Exposure to antibiotics during pregnancy or early infancy and the risk of autoimmune disease in children: A nationwide cohort study in Korea. *PLoS Med*. 2025;22(8):e1004677. Published 2025 Aug 21. doi:10.1371/journal.pmed.1004677
103. Flöistrup H, Swartz J, Bergström A, et al. Allergic disease and sensitization in Steiner school children. *J Allergy Clin Immunol*. 2006;117(1):59-66. doi:10.1016/j.jaci.2005.09.039
104. Cait A, Wedel A, Arntz JL, et al. Prenatal antibiotic exposure, asthma, and the atopic march: A systematic review and meta-analysis. *Allergy*. 2022;77(11):3233-3248. doi:10.1111/all.15404
105. Baron R, Taye M, der Vaart IB, et al. The relationship of prenatal antibiotic exposure and infant antibiotic administration with childhood allergies: a systematic review. *BMC Pediatr*. 2020;20(1):312. Published 2020 Jun 27. doi:10.1186/s12887-020-02042-8
106. Cui H, Mu Z. Prenatal Maternal Risk Factors Contributing to Atopic Dermatitis: A Systematic Review and Meta-Analysis of Cohort Studies. *Ann Dermatol*. 2023;35(1):11-22. doi:10.5021/ad.21.268
107. Gestels T, Vandenplas Y. Prenatal and Perinatal Antibiotic Exposure and Long-Term Outcome. *Pediatr Gastroenterol Hepatol Nutr*. 2023;26(3):135-145. doi:10.5223/pghn.2023.26.3.135
108. Huang FQ, Lu CY, Wu SP, Gong SZ, Zhao Y. Maternal exposure to antibiotics increases the risk of infant eczema before one year of life: a meta-analysis of observational studies. *World J Pediatr*. 2020;16(2):143-151. doi:10.1007/s12519-019-00301-y
109. Tsakok T, McKeever TM, Yeo L, Flohr C. Does early life exposure to antibiotics increase the risk of eczema? A systematic review. *Br J Dermatol*. 2013;169(5):983-991. doi:10.1111/bjd.12476
110. Wan M, Yang X. Maternal exposure to antibiotics and risk of atopic dermatitis in childhood: a systematic review and meta-analysis. *Front Pediatr*. 2023;11:1142069. Published 2023 May 15. doi:10.3389/fped.2023.1142069
111. Zhong Y, Zhang Y, Wang Y, Huang R. Maternal antibiotic exposure during pregnancy and the risk of allergic diseases in childhood: A meta-analysis. *Pediatr Allergy Immunol*. 2021;32(3):445-456. doi:10.1111/pai.13411
112. Carson CG. Risk factors for developing atopic dermatitis. *Dan Med J*. 2013;60(7):B4687.
113. Milliken S, Allen RM, Lamont RF. The role of antimicrobial treatment during pregnancy on the neonatal gut microbiome and the development of atopy, asthma, allergy and obesity in childhood. *Expert Opin Drug Saf*. 2019;18(3):173-185. doi:10.1080/14740338.2019.1579795
114. Wrześniewska M, Wołoszczak J, Świrkosz G, Szyller H, Gomułka K. The Role of the Microbiota in the Pathogenesis and Treatment of Atopic Dermatitis-A Literature Review. *Int J Mol Sci*. 2024;25(12):6539. Published 2024 Jun 13. doi:10.3390/ijms25126539
115. Grijincu M, Buzan MR, Zbîrcea LE, Păunescu V, Panaitescu C. Prenatal Factors in the Development of Allergic Diseases. *Int J Mol Sci*. 2024;25(12):6359. Published 2024 Jun 8. doi:10.3390/ijms25126359
116. Zhang MZ, Chu SS, Xia YK, Wang DD, Wang X. Environmental exposure during pregnancy and the risk of childhood allergic diseases. *World J Pediatr*. 2021;17(5):467-475. doi:10.1007/s12519-021-00448-7
117. Wang L, Xu L. The impact of prebiotics, probiotics and synbiotics on the prevention and treatment of atopic dermatitis in children: an umbrella meta-analysis. *Front Pediatr*. 2025;13. doi:10.3389/fped.2025.1498965
118. Greenzaid J, Chan L, Chandani B, Kiritsis N, Feldman S. Microbiome modulators for atopic eczema: a systematic review of experimental and investigational therapeutics. *EXPERT Opin Investig DRUGS*. 2024;33(4):415-430. doi:10.1080/13543784.2024.2326625
119. El-Heis S, Crozier SR, Inskip HM, Harvey NC, Healy E, Godfrey KM. Early life exposure to antibiotics and laxatives in relation to infantile atopic eczema. *JOURNAL OF INVESTIGATIVE DERMATOLOGY*. 2022;142(12, S):S193.
120. Davis KL. Pre- and post-natal exposure to antibiotics and the development of eczema, recurrent wheezing, and atopic sensitization in children up to the age of 4 years. *Pediatrics*. 2011;128(SUPPL. 3):S96. doi:10.1542/peds.2011-2107F
121. Chen Y, Li SM, Yau JW, et al. Perinatal factors for resolution of early-onset eczema by one year old: A Chinese birth cohort in Hong Kong. *Allergy Eur J Allergy Clin Immunol*. 2020;75(SUPPL 109):439. doi:10.1111/all.14508.
122. Li Y, Liu LH, Jian ZY, et al. Association between antibiotic exposure and adverse outcomes of children and pregnant women: evidence from an umbrella review. *World J Pediatr*. 2023;19(12):1139-1148. doi:10.1007/s12519-023-00711-z
123. Ohya Y, Nomura I, Natori M, et al. Antibiotics during pregnancy is a risk factor for child’s eczema and wheezing. *JOURNAL OF ALLERGY AND CLINICAL IMMUNOLOGY*. 2008;121(2, 1):S238. doi:10.1016/j.jaci.2007.12.941
124. Stefanaki E, Kalaitzidou I, Aristou M, Lakoumentas J, Galanakis E, Xepapadaki P. Prenatal antibiotics and atopic dermatitis among 18-month old children in Crete, Greece. *Allergy Eur J Allergy Clin Immunol*. 2021;76(SUPPL 110):574. doi:10.1111/all.15096
125. Tsakok T, McKeever TM, Yeo L, Flohr C. Does early life exposure to antibiotics increase the risk of eczema? A systematic review. *Br J Dermatol*. 2013;169(5):983-991. doi:10.1111/bjd.12476
126. Tsakok T, McKeever T, Yeo L, Flohr C. Early life antibiotic exposure and the risk of atopic eczema. *Br J Dermatol*. 2012;167(2):e9. doi:10.1111/j.1365-2133.2012.011134.x
127. Zeng X, Zhao W, Li CC, Huang L, Zhang WX. Maternal Antibiotic Exposure is Associated with Childhood Atopic Dermatitis. *J Allergy Clin Immunol*. 2020;145(2):AB192. doi:10.1016/j.jaci.2019.12.272

**Supplementary Table 2:** Additional population data regarding the prenatal group

| Author name, year | Exposure measurement | Outcome measurement | Mother exposed to antibiotics | Mother not exposed to antibiotics | Number of children involved | Total affected children | Adjusted confounders |
| --- | --- | --- | --- | --- | --- | --- | --- |
| Ahmad, 2021 | Questionnaire | Questionnaire | 476 | 4009 | 2960 | NA | Age of the mother; gender of the child; whether English is spoken at home; maternal relationship status; indigenous status; education of the mother; Socio-economic status; remoteness of the family residence; type of birth; birthweight; the immunisation status of the child; mother's quality of sleep one year before birth; home exterior condition |
| Bisgaard, 2009 | Clinical research unit | Clinical research unit | 49 | 307 | 356 | 134 | Mother’s eczema; Mother's work; Filaggrin mutations; Alcohol, 3rd trimester; Exercise per week 3rd trimester; Length of child; Dog at home; Breastfeeding (solely); Temperature first year |
| Chang, 2023 | Database | Database | 508647 | 779696 | 1288343 | 286772 | Maternal age; urbanization; insurance property; frequency of outpatient clinic visits; mode of delivery; type of pregnancy; acetaminophen use during pregnancy; maternal atopic disorders; gestational infections; maternal comorbidities; birth year; child's sex; length of gestation; birth weight; APGAR score at birth |
| Choi, 2025 | Database | Database | 13932 | 68470 | 82402 | 27228 | No information |
| Dom, 2011 | Questionnaire | Questionnaire, blood sample | 154 | 619 | 773 | 243 | Maternal age, infant's Gender, daycare attendance, breastfeeding, prenatal exposure to cats or dogs, postnatal exposure to cats or dogs, prenatal exposure to cigarette smoke, postnatal exposure to cigarette smoke, parental education, number of siblings, birth weight, lower respiratory tract infections, parental history of allergies |
| El-Heis, 2023 | Interview | Questionnaire , nurses examined | NA | NA | 3158 | 270 | Maternal BMI; parity; breastfeeding duration; infant sex; maternal education |
| Fuxench, 2024 | Database | Database | 185291 | 837849 | 1023140 | 259059 | No information |
| Gao, 2019 | Questionnaire | Questionnaire, interview | 15 | 888 | 903 | 226 | Antibiotic exposure in first year of life, parental history of allergy, eggs consumption, milk or milk products consumption, infant sex |
| Hesla, 2017 | Parent report | Parent reported | 69 | 390 | 490 | 55 | No information |
| Jedrychowsky, 2006 | Questionnaire | (Questionnaire,) interview | 31 | 71 | 102 | 34 | Maternal education level; child's gender; respiratory infections over the pregnancy; maternal allergy; maternal age; environmental tobacco smoke; paternal allergy |
| Kelderer, 2021 | Questionnaire | Parent reported | 114 | 1126 | 1240 | 424 | Infant's sex; breastfeeding at 4 months; maternal history of allergic disease; paternal history of allergic disease; exposure to pets |
| Kurzius-Spencer, 2004 | Questionnaire | Questionnaire, medical records | 70 | 290 | 364 | 101 | Infant sex; maternal asthma during pregnancy; birth order; paternal asthma; maternal prenatal total IgE; eczema during pregnancy; maternal cold in 3rd trimester; maternal prenatal IL-10 production |
| Lee, 2014 | Medical record | Clinical diagnosis | 38 | 346 | 412 | 116 | Gestational age at birth; sex; pre-pregnancy BMI; maternal age at delivery; maternal educational level; prenatal exposure to smoke; prenatal exposure to pets; presence of older siblings; parental allergy disease history |
| Lin, 2022 | Database | Database | 7193 | 14623 | 21817 | 5454 | Age (infant); Infant gender; congenital anomalies of the heart; maternal age at birth; maternal atopy; child's infection; neonatal hiperbilirubinaemia; prior atopic comorbidities; Mode of delivery; hypertension; gestational diabetes mellitus; preterm birth; Kawasaki disease; epilepsy; icthyosis; respiratory distress syndrome |
| McKeever, 2002 | Database | Database | 8676 | 16014 | 24690 | 7758 | Maternal smoking; Maternal age; number of older siblings; year of birth; general practice; maternal allergic disease; consulting behaviour; Gender; Antibiotics in first months of life |
| Metzler, 2019 | Questionnaire | Parent reported | 288 | 792 | 1080 | 288 | Parental atopic status; gender; smoking during pregnancy; number of siblings; environment (rural or centre); pets (dogs and cats) during pregnancy, mode of delivery; maternal education |
| Mubanga, 2021 | Medical report | Medical report | 153407 | 569360 | 722767 | 82274 | Age; Sex; Mother's age; Family situation; Parity; Level of education; area of residence; smoking history; maternal history of asthma |
| Okoshi, 2023 | Questionnaire+ medical report | Questionnaire | 22433 | 56245 | 78678 | 7067 | Maternal age at delivery; parity; marital status; prepregnancy body mass index; preexisting hypertension; preexisting diabetes; maternal history of allergies; antipyretic or analgesic use during pregnancy; maternal education; household income; complication of pregnancy or delivery; morning sickness; weight gain during pregnancy; urinary cotinine concentration during pregnancy; alcohol consumption during pregnancy; sex of the infant; premature birth; birth weight; breastfeeding; pet ownership |
| Panduru, 2020 | Questionnaire | Questionnaire | 115,9112 | 930,0888 | 1046 | 117 | No information |
| Sariachvili, 2007 | Questionnaire | Questionnaire (parent report) | 183 | 769 | 976 | 227 | Maternal atopy; paternal atopy; pregnancy duration; maternal educational level; maternal age; active and passive smoking during pregnancy; passive smoking for the child in 1st year of life; gender; postnatal contact with pets; parity; daycare attendance; use of antibiotics in first year of life |
| Sasaki, 2019 | Interview, medical records | Questionnaire on clinical diagnosis | 20869 | 49539 | 69324 | 12702 | Maternal education; maternal smoking during pregnancy; maternal history of asthma,AD and allergic rhinits; child's sex |
| Stefanaki, 2023 | Interview | Interview | 55 | 176 | 236 | 43 | No information |
| Tai, 2024 | Database | Database | 484202 | 422740 | 900584 | 249094 | Maternal age; mode of delivery; maternal comorbidities; maternal allergic diseases; pregnancy-related complications; infants’ gender |
| Timm, 2017 | Interview | Parent reported | ? | 49872 | 62560 | 8733 | Maternal prenatal smoking; Maternal atopy; Household socio-economic status; Older siblings; Mode of delivery |
| Vance, 2023 | Questionnaire | Self report | 1159 | 7935 | 9094 | 1083 | Maternal age at return of maternal questionnaire; mother’s age of delivery; maternal body mass index; smoking status during pregnancy; Race; birthweight; parental history of allergies |

**Supplementary Table 3.** Additional population data regarding the intrapartum group

| **Author name, *year*** | **Exposure measurement** | **Outcome measurement** | **Mother exposed to antibiotics** | **Mother not exposed to antibiotics** | **Number of children involved** | **Total affected children** | **Adjusted confounders** |
| --- | --- | --- | --- | --- | --- | --- | --- |
| Dhudasia, 2021 | Medical report | Medical report | 7057 | 6989 | 14 046 | 1331 | BMI of mother; parity; maternal history of asthma or allergy; GBS colonization; diagnosis of intraamniotic infection; Infant's sex; birth weight; socioeconomic status; Neonatal antibiotics; Breastfeeding |
| Hong, 2022 | Questionnaire+medical records | Questionnaire | 284 | 2625 | 2909 | 397 | Maternal age, Maternal height; Parity, Mode of delivery, previous GBS colonization, Maternal allergies, Smoking history, Pet ownership, Resident place, Education level, Feeding patterns, Antibiotic use in first 72h of life, Infant major anomalies, Maternal neuropsychiatric disorders |
| Puisto, 2022 | Study records | Clinical diagnosis | 63 | 357 | 433 | 166 | Child's sex; Older siblings; Smoking during pregnancy; Breastfeeding duration |
| Wohl, 2015 | Medical report | Medical report | 128 | 364 | 492 | 137 | No information |


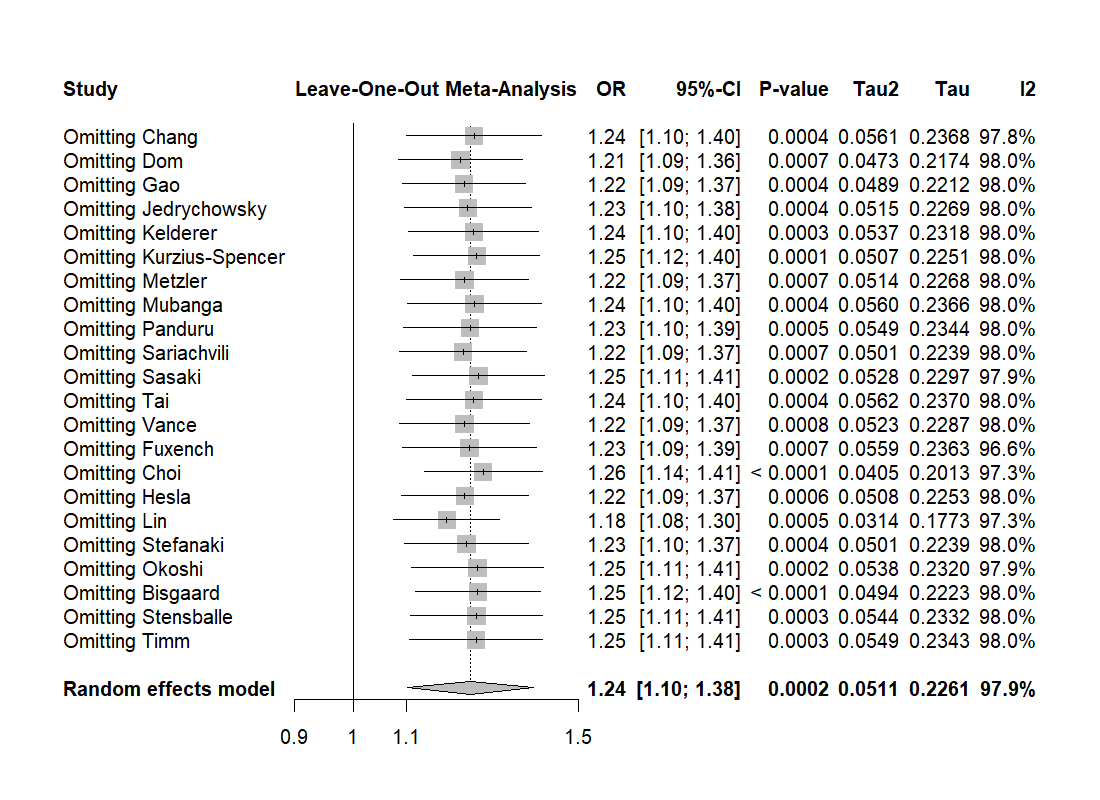
 **Supplementary Figure 1.** The leave out one analysis of the prenatal antibiotic exposre studies regarding OR data


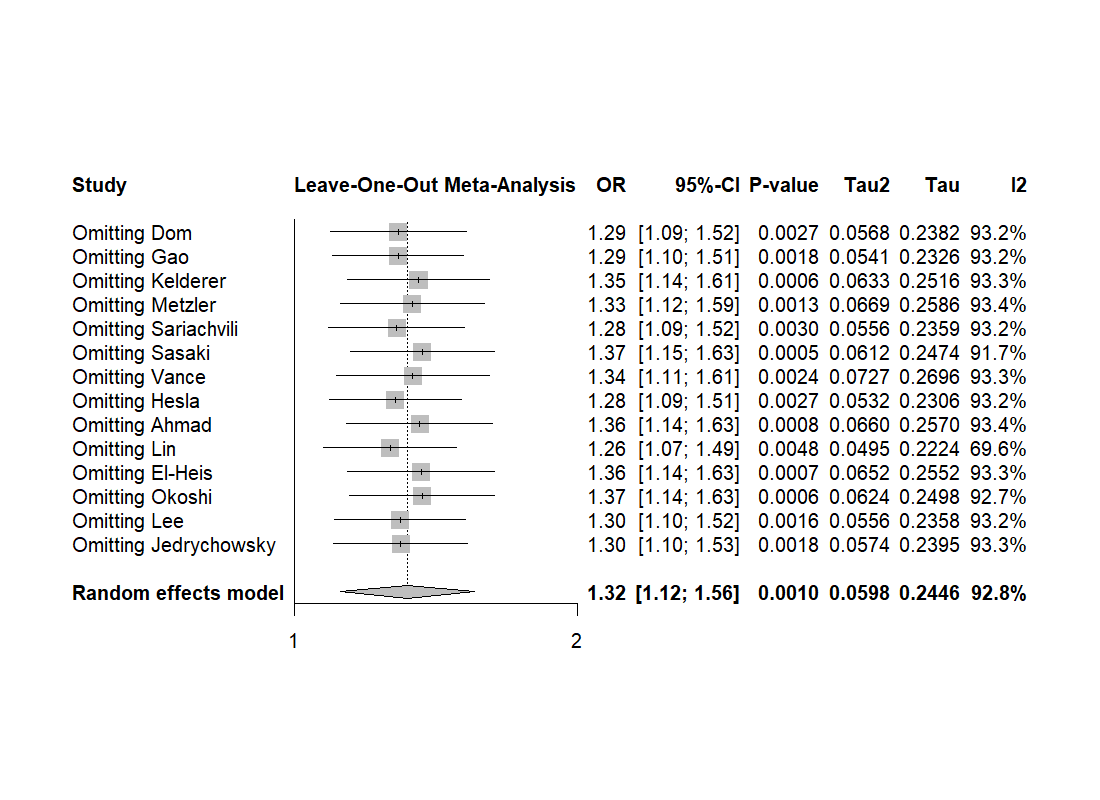


**Supplementary Figure 2.** The leave out one analysis of the prenatal antibiotic exposre studies regarding aOR data


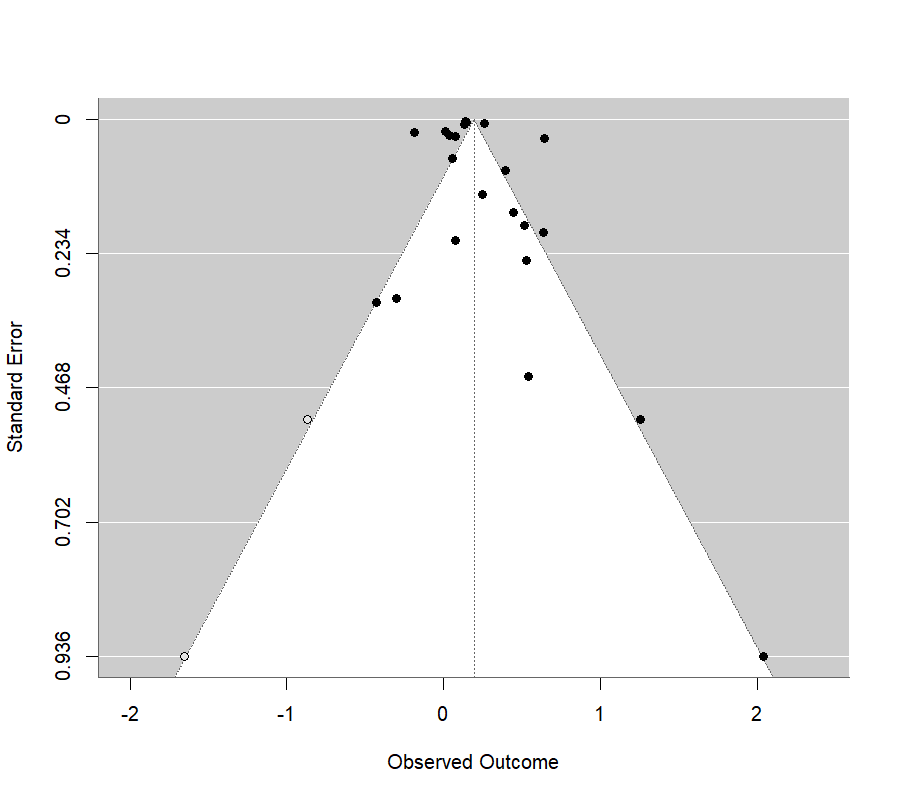


**Supplementary Figure 3.** The funnel plot of the prenatal antibiotic exposre studies regarding OR data


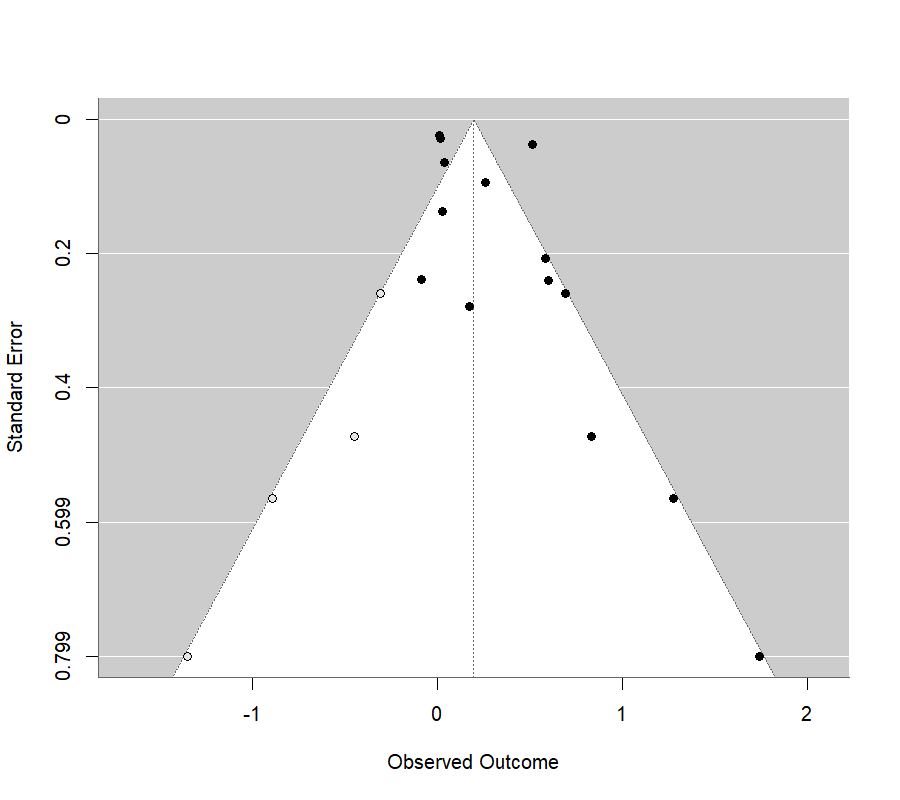


**Supplementary Figure 4.** The funnel plot of the prenatal antibiotic exposre studies regarding aOR data


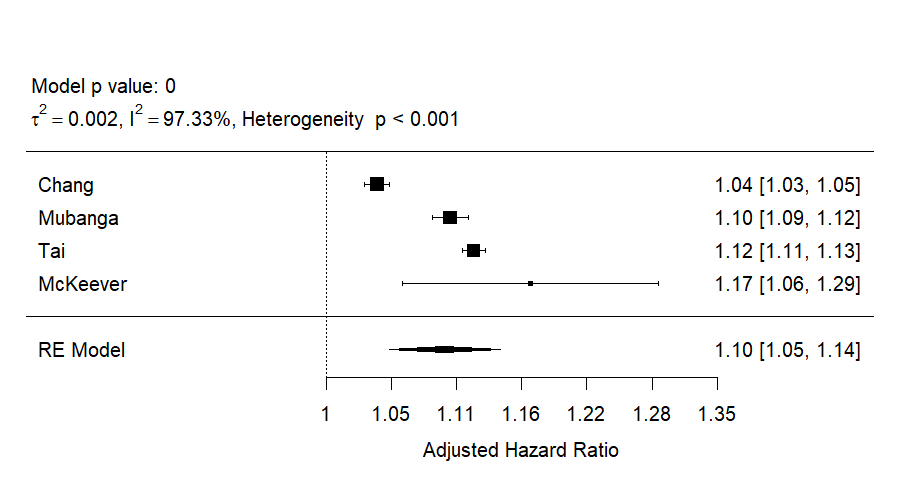


**Supplementary Figure 5.** The effect size of prenatal antibiotic exposure on childhood eczema based on aHR data


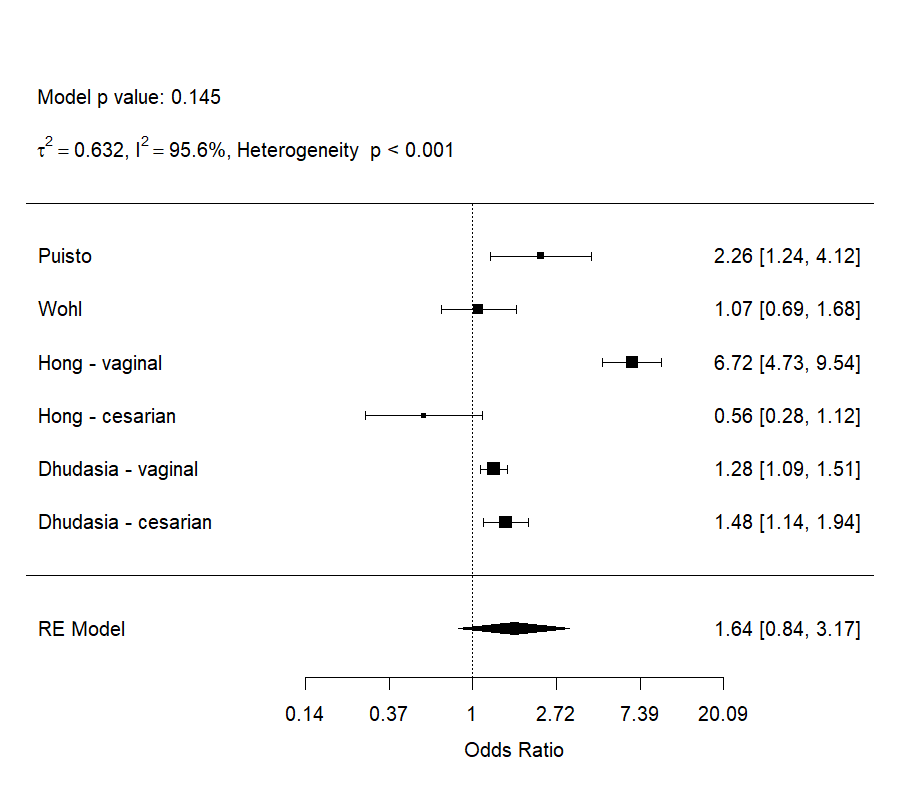


**Supplementary Figure 6.** The effect size of intrapartum antibiotic exposure on childhood eczema regarding OR data


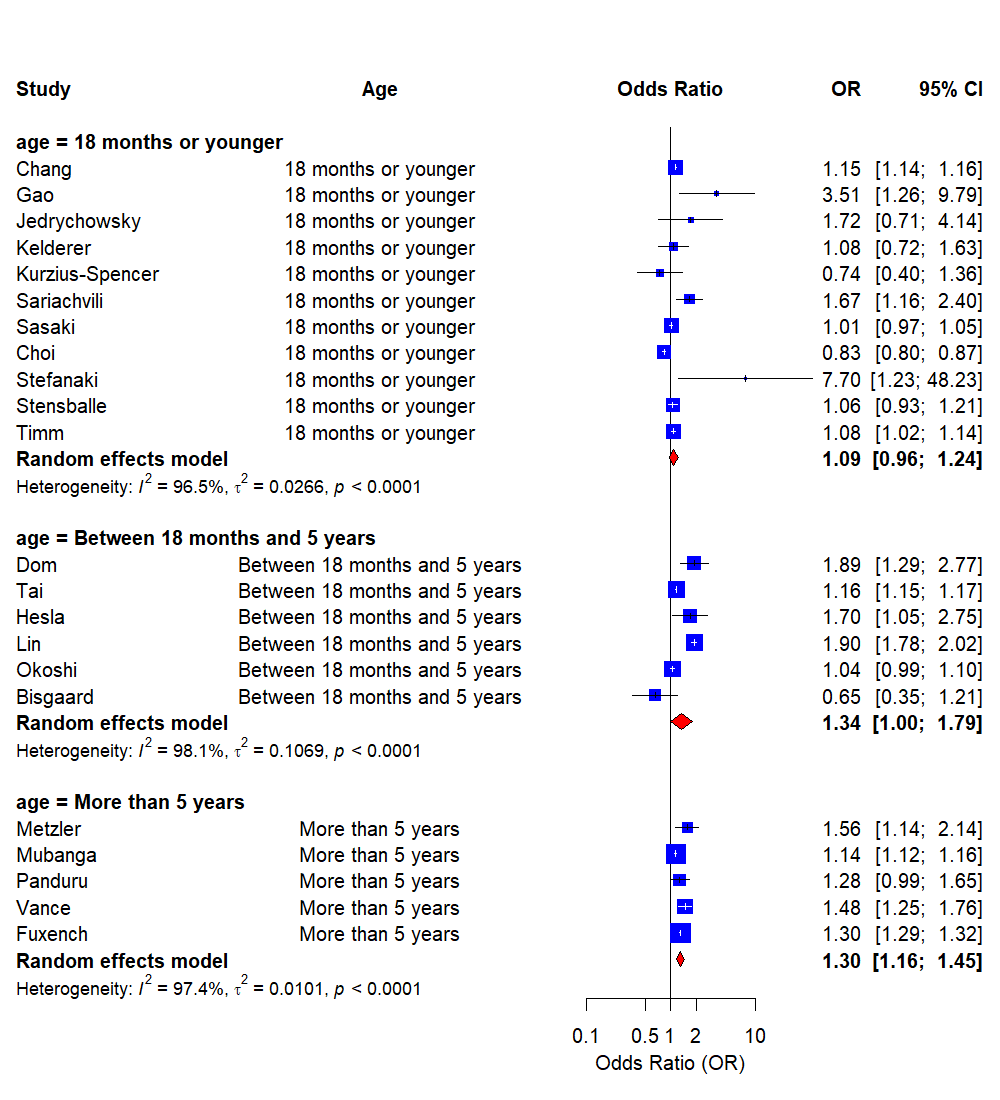


**Supplementary Figure 7.** OR data regarding the age of participants in the prenatal antibiotic exposure group


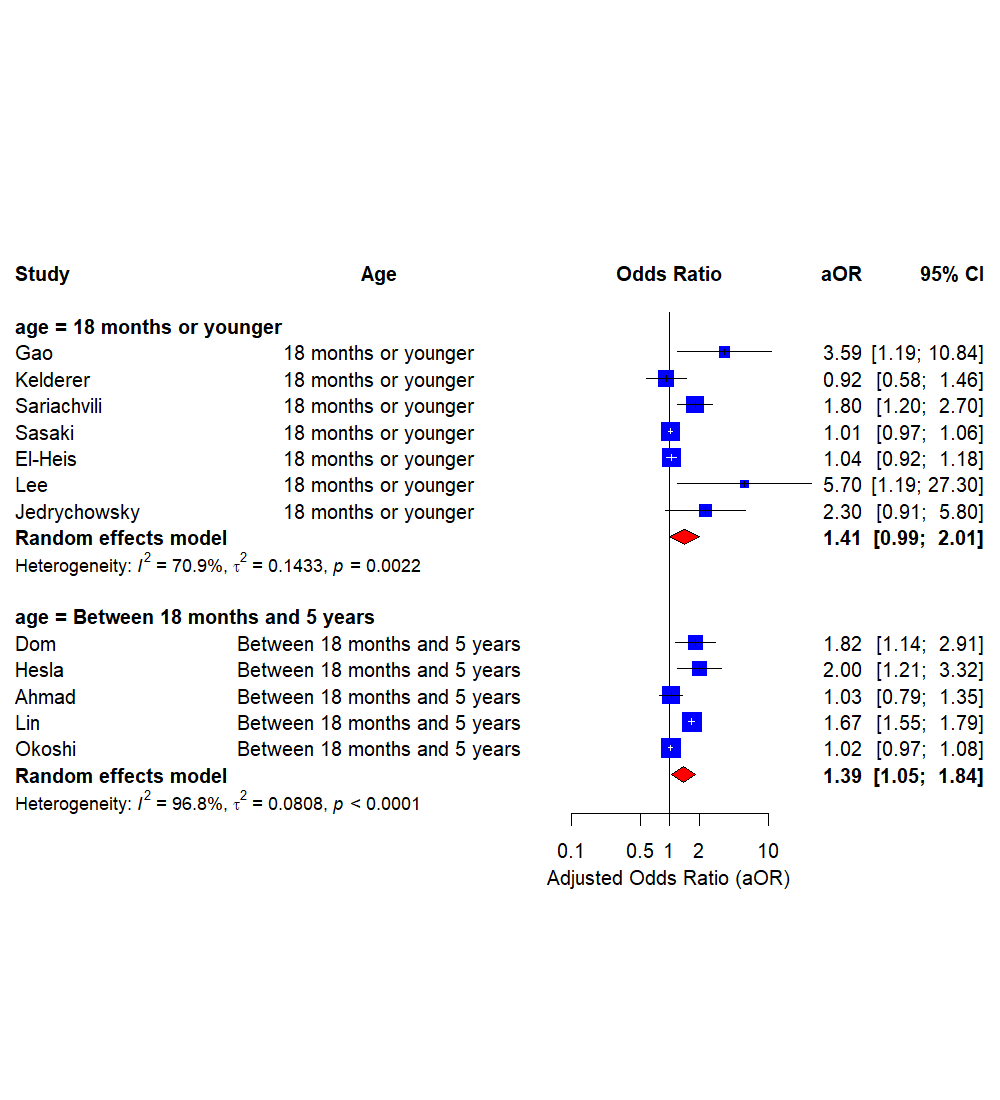


**Supplementary Figure 8**. The aOR data regarding the age of participants in the prenatal antibiotic exposure group


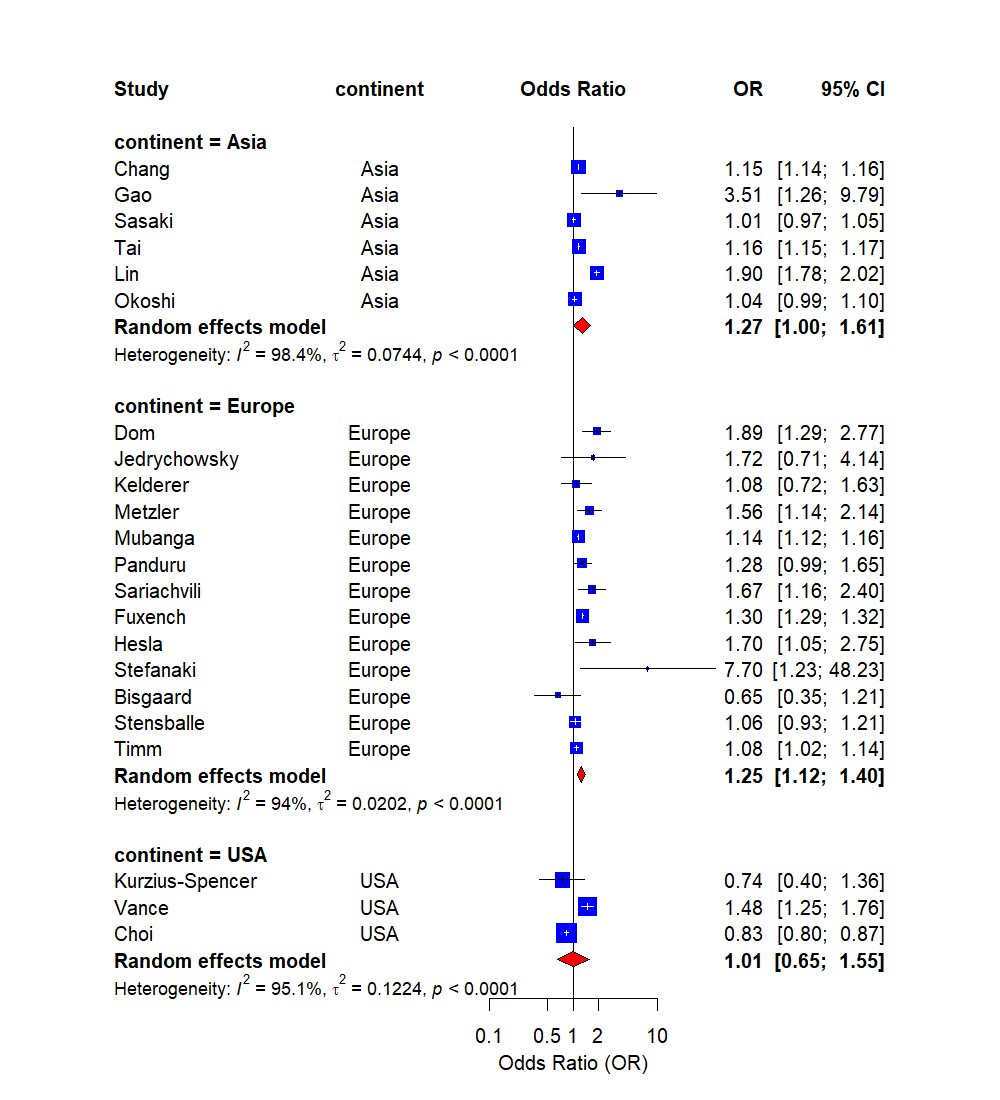


**Supplementary Figure 9.** The OR data regarding the origin of the studies in the prenatal antibiotic exposure group


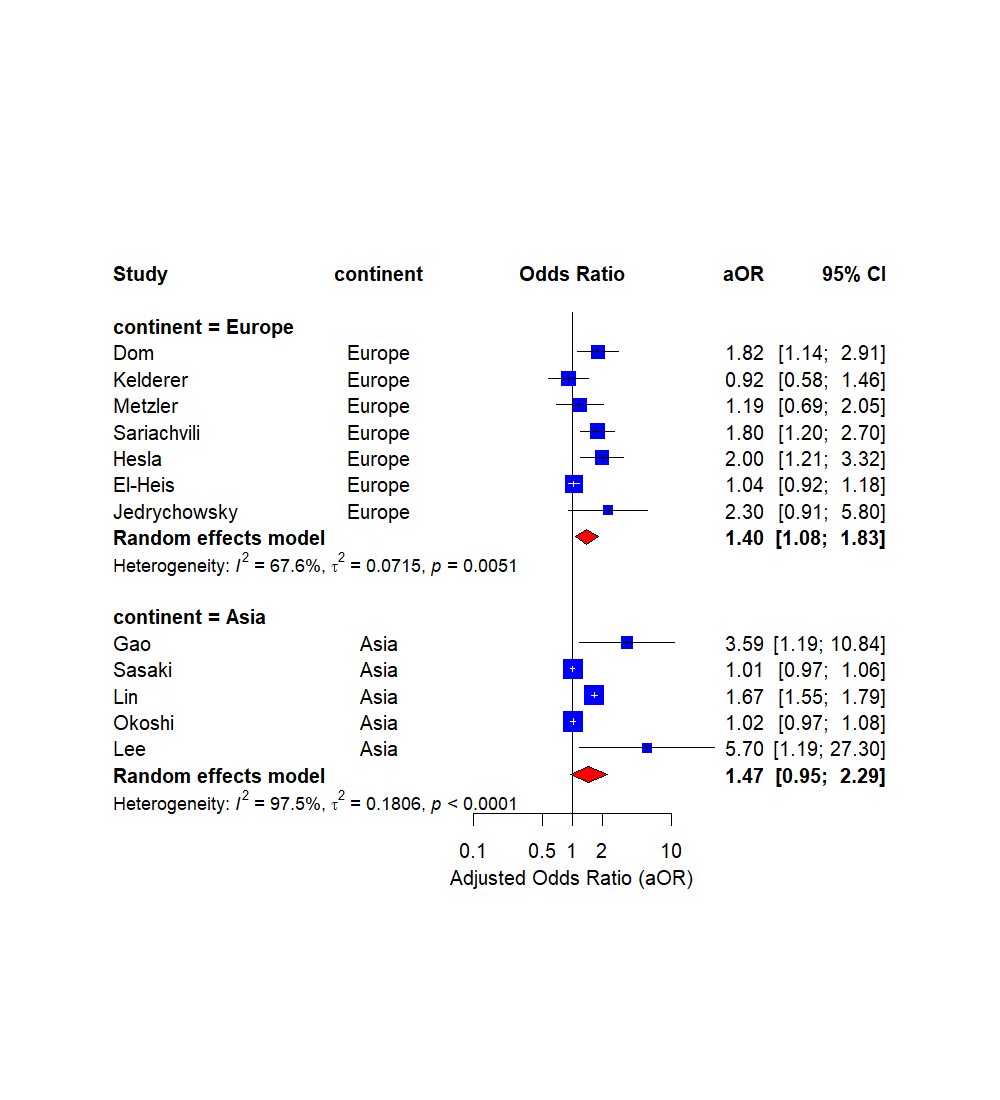


**Supplementary Figure 10.** The aOR data regarding the origin of the studies in the prenatal antibiotic exposure group


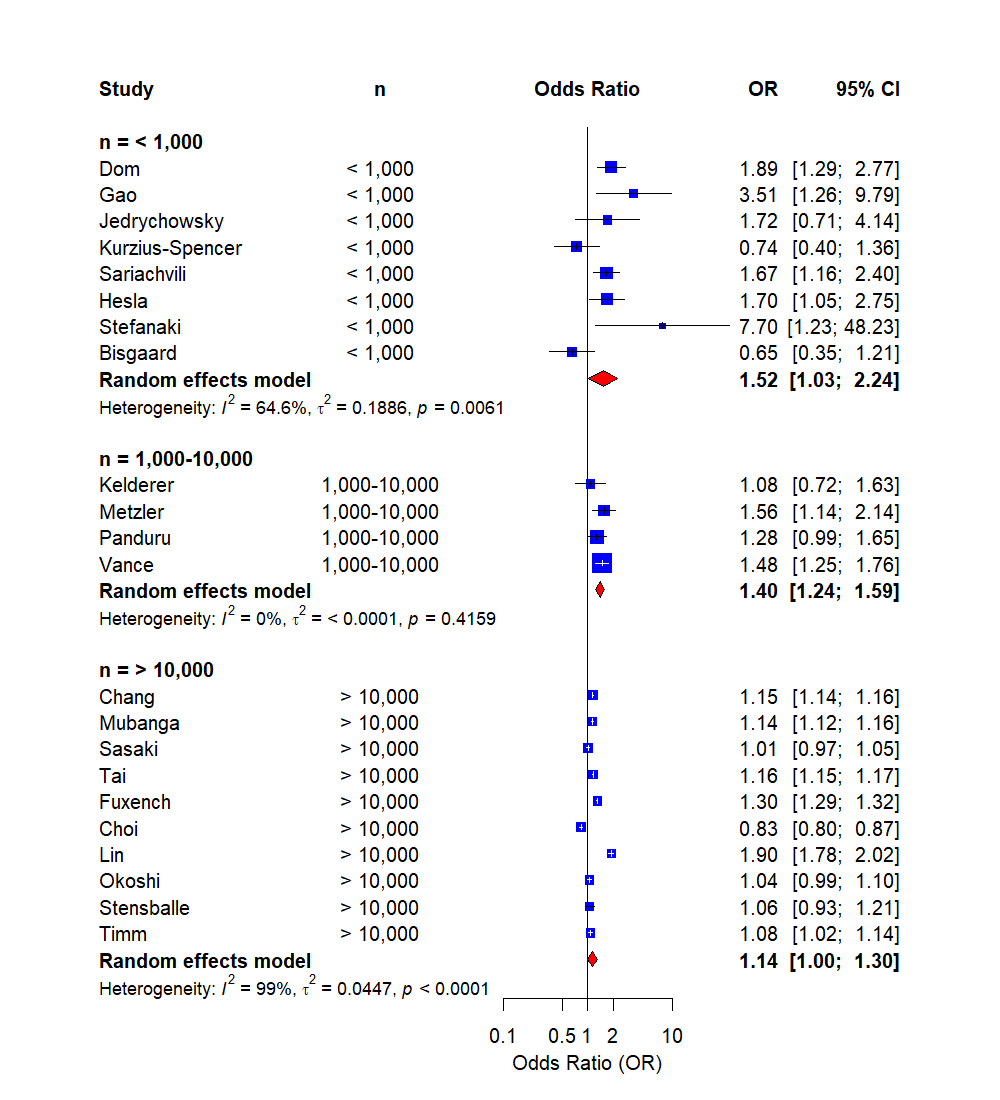


**Supplementary Figure 11.** The OR data regarding the number of participants in the prenatal antibiotic exposure group


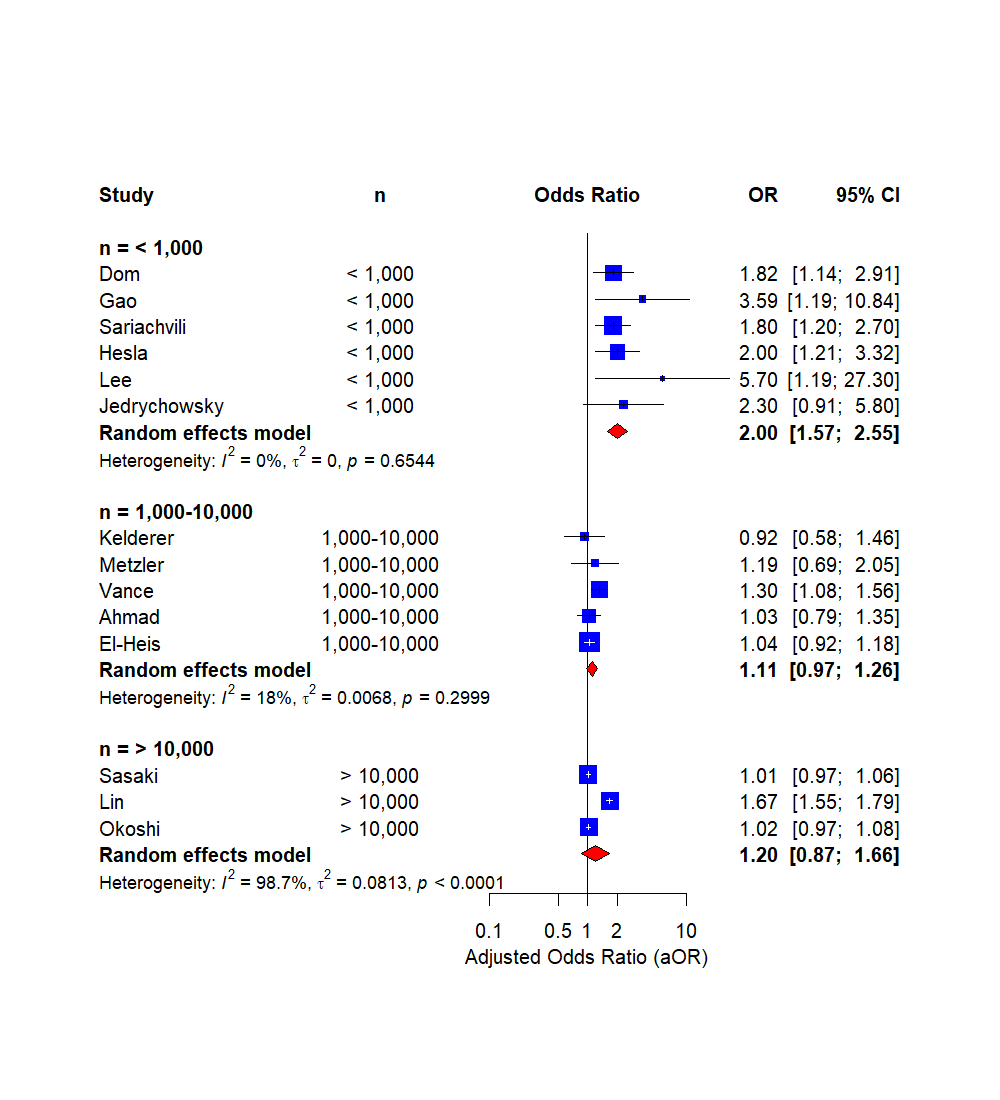


**Supplementary Figure 12**. The aOR data regarding the number of participants in the prenatal antibiotic exposure group


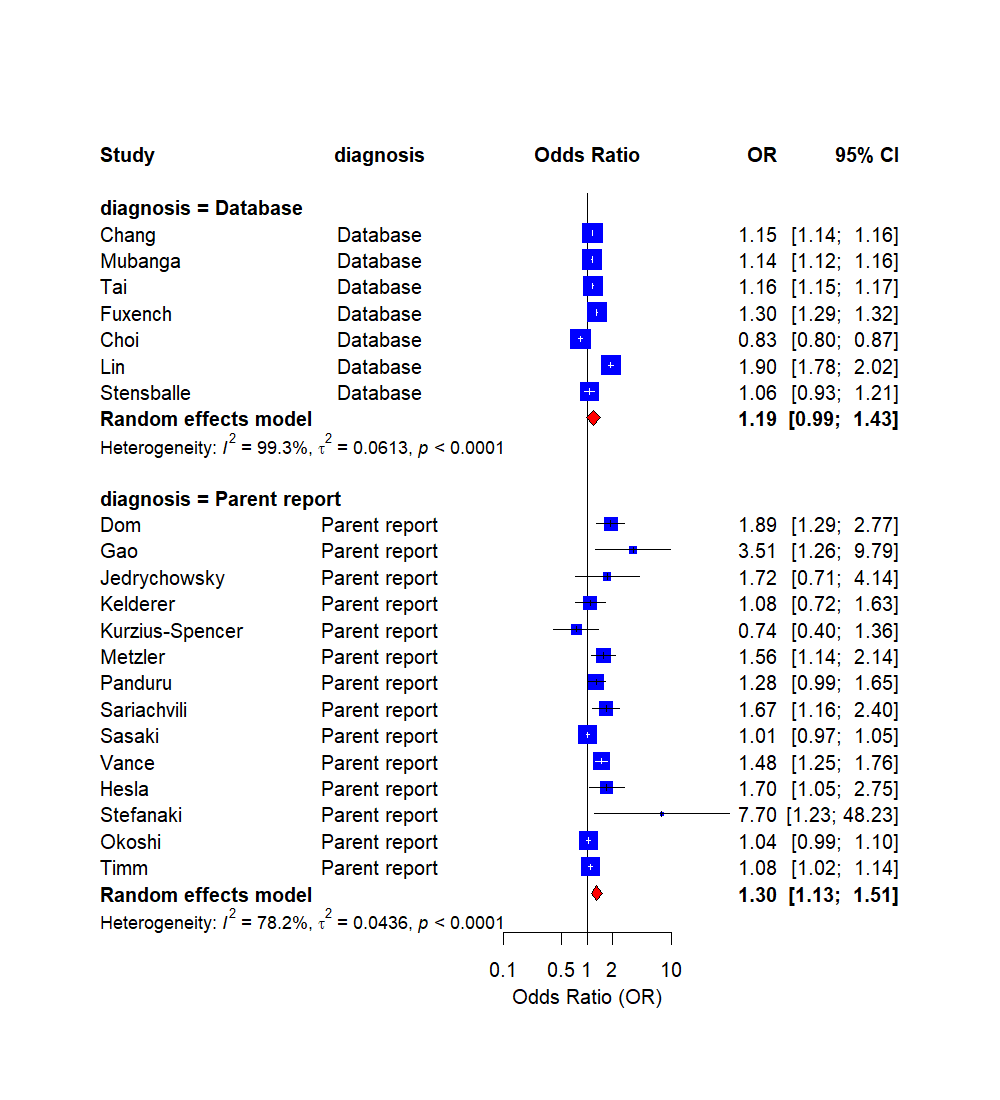


**Supplementary Figure 13.** OR data regarding mode of diagnosis in the prenatal antibiotic exposure group


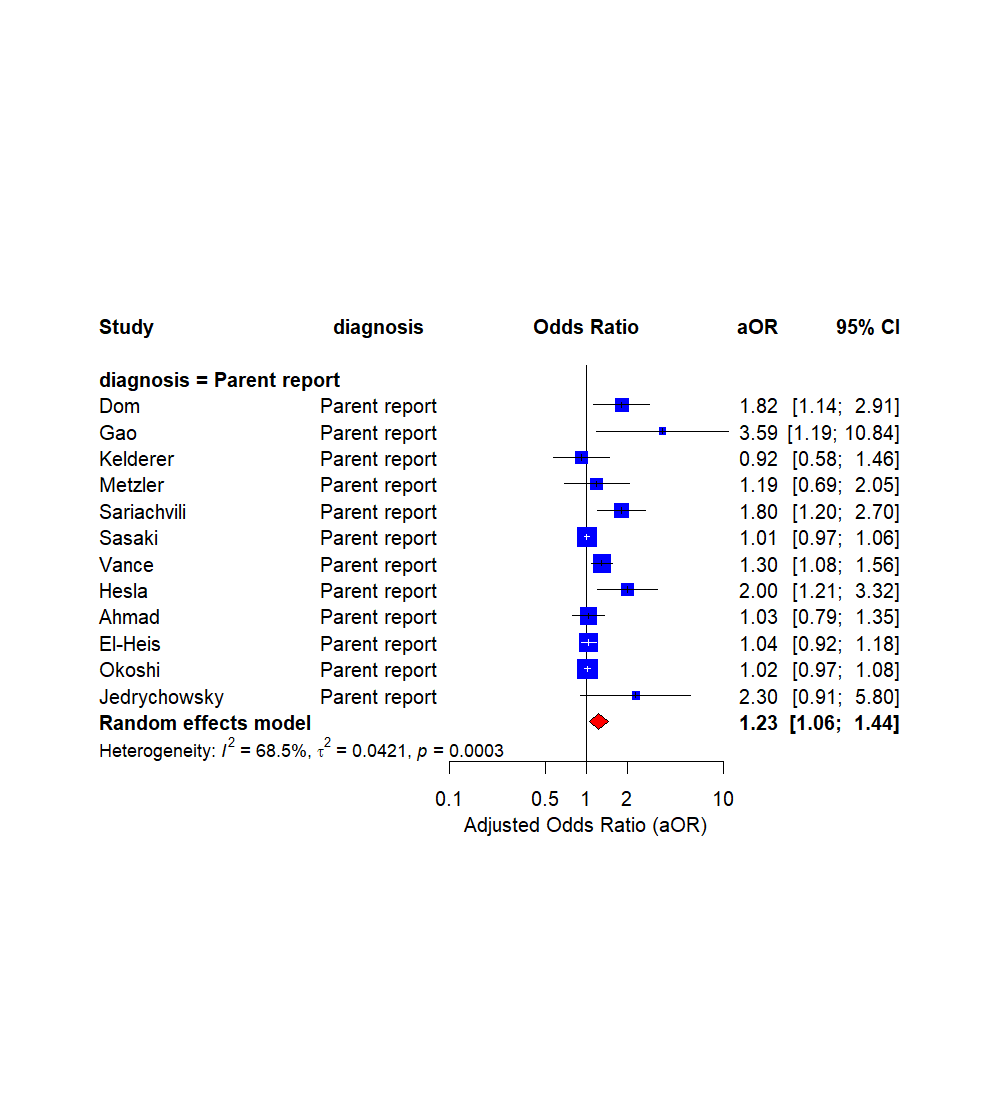


**Supplementary Figure 14.** aOR data regarding mode of diagnosis in the prenatal antibiotic exposure group


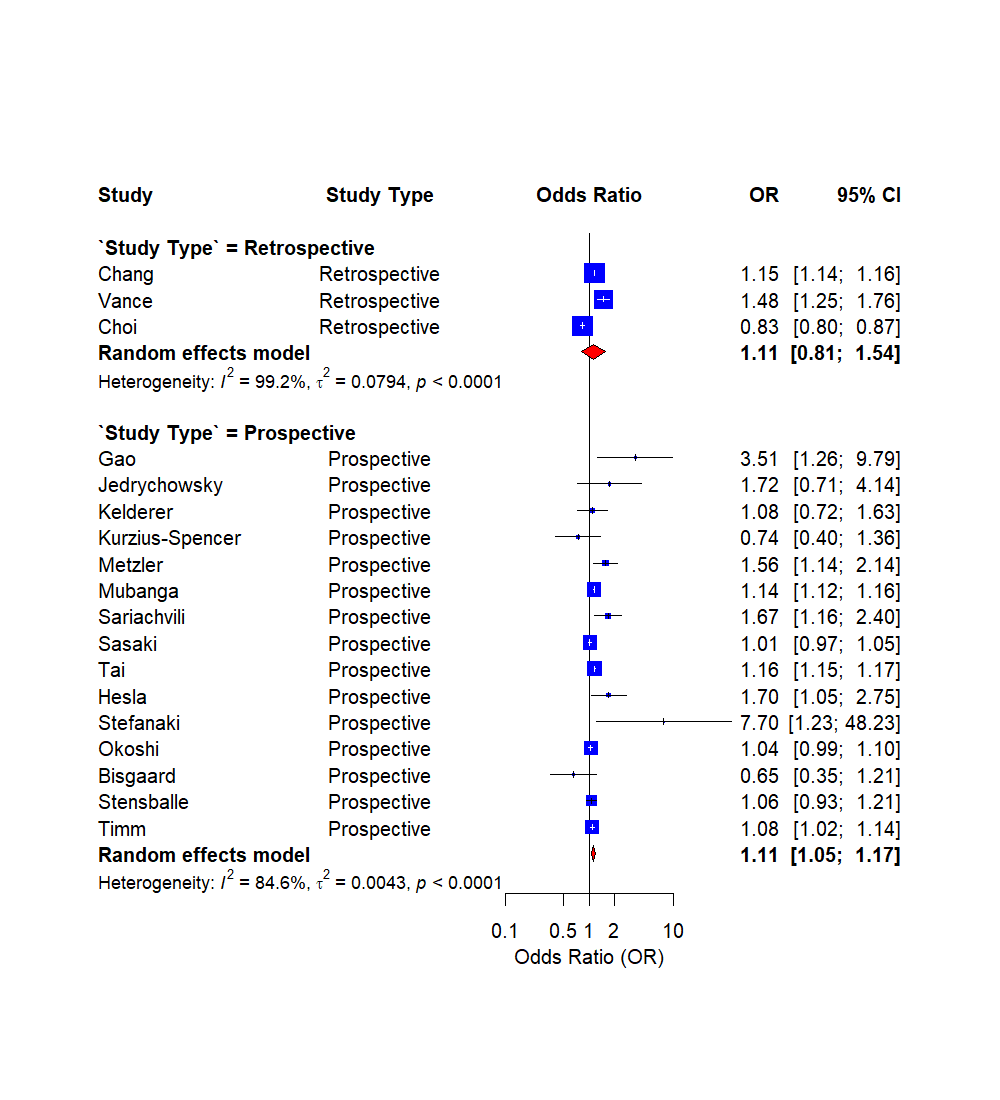


**Supplementary Figure 15.** The OR data regarding study type in the prenatal antibiotic exposure group


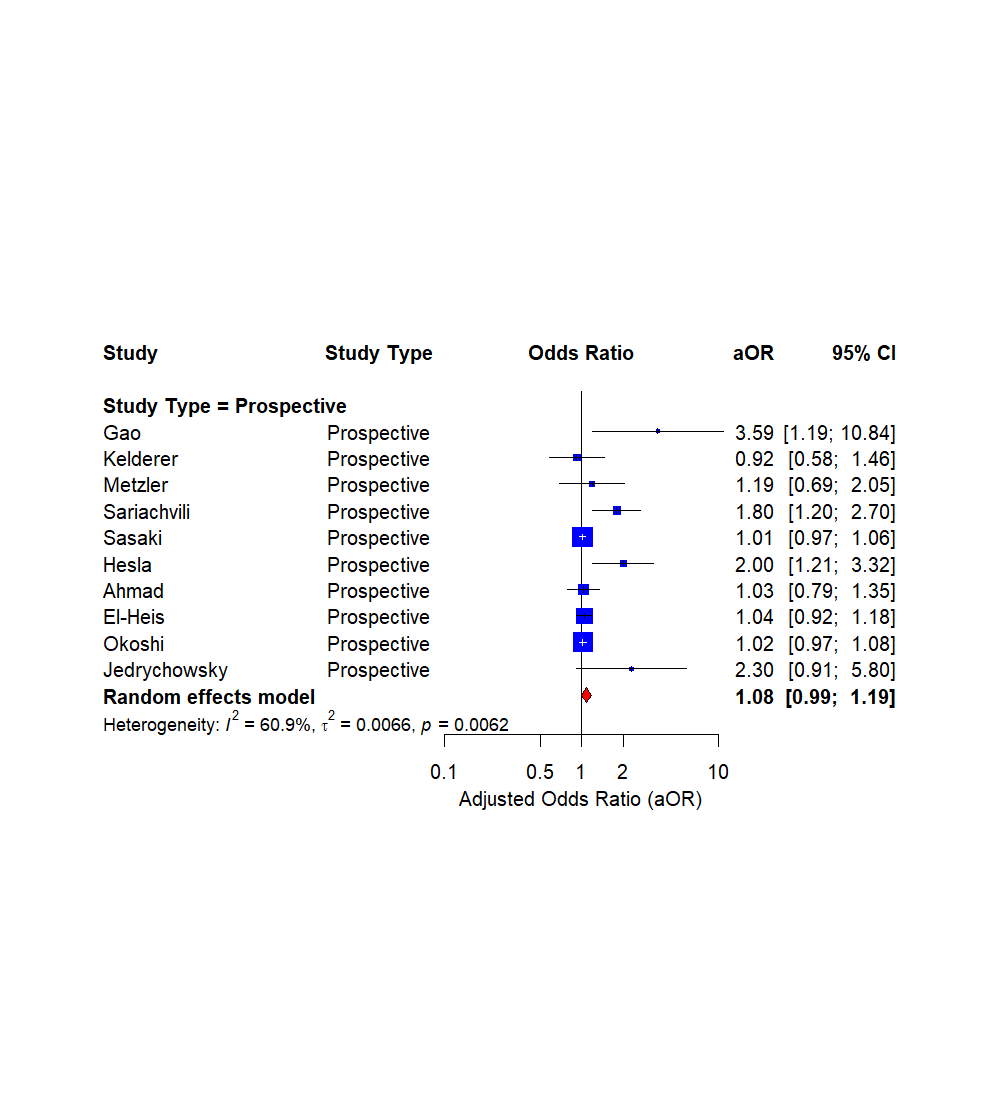


**Supplementary Figure 16.** The aOR data regarding study type in the prenatal antibiotic exposure group


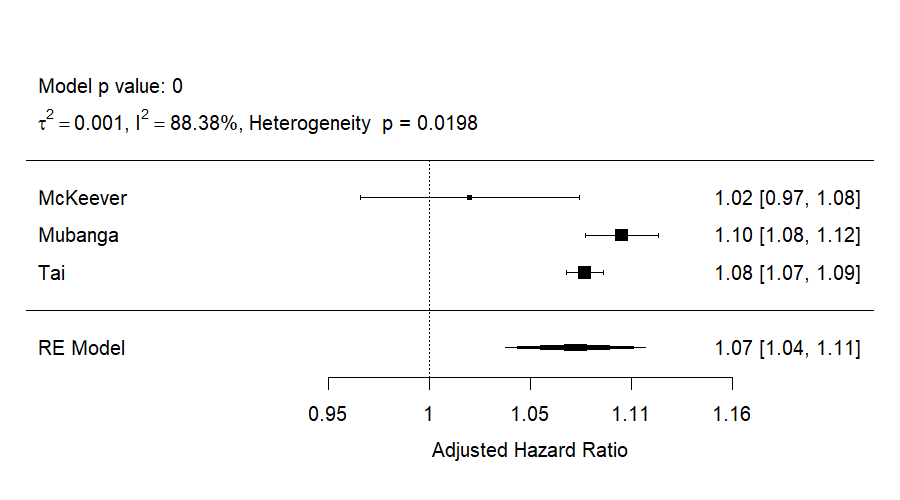


**Supplementary Figure 17.** The aHR data of antibiotic use once only in the prenatal antibiotic exposure group


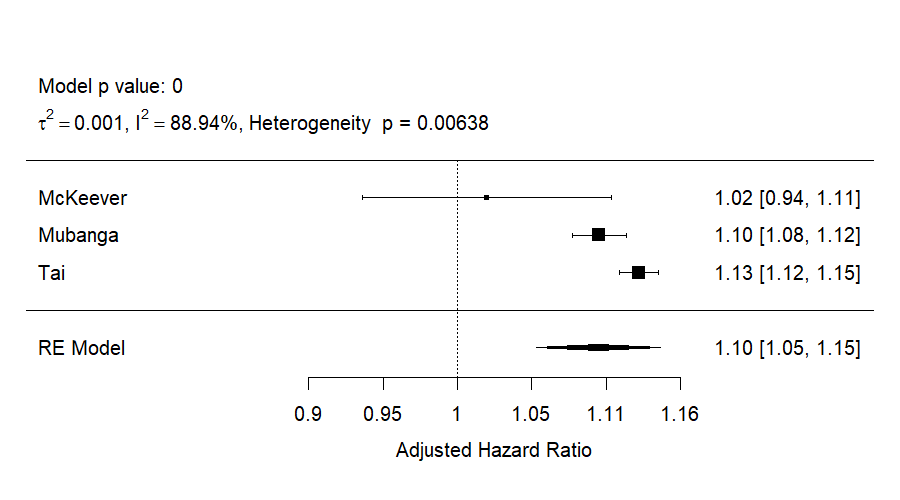


**Supplementary Figure 18.** The aHR data of antibiotic use twice in the prenatal antibiotic exposure group


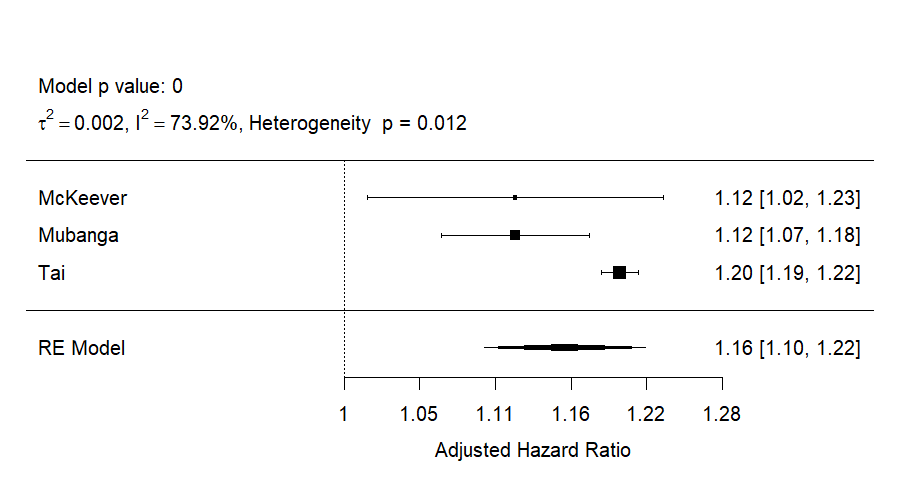


**Supplementary Figure 19.** The aHR data of antibiotic use more than two times in the prenatal antibiotic exposure group

**Supplementary Table 4**: The list of studies included in the traffic light plots

| Study number | First author |
| --- | --- |
| 1 | Ahmad |
| 2 | Bisgaard |
| 3 | Chang |
| 4 | Choi |
| 5 | Dom |
| 6 | El-Heis |
| 7 | Fuxench |
| 8 | Gao |
| 9 | Hesla |
| 10 | Jedrychowski |
| 11 | Kelderer |
| 12 | Kurzius-Spencer |
| 13 | Lee |
| 14 | Lin |
| 15 | McKeever |
| 16 | Metzler |
| 17 | Mubanga |
| 18 | Okoshi |
| 19 | Panduru |
| 20 | Sariachvili |
| 21 | Sasaki |
| 22 | Stefanaki |
| 23 | Stensballe |
| 24 | Tai |
| 25 | Timm |
| 26 | Vance |
| 27 | Dhudasia |
| 28 | Hong |
| 29 | Puisto |
| 30 | Wohl |


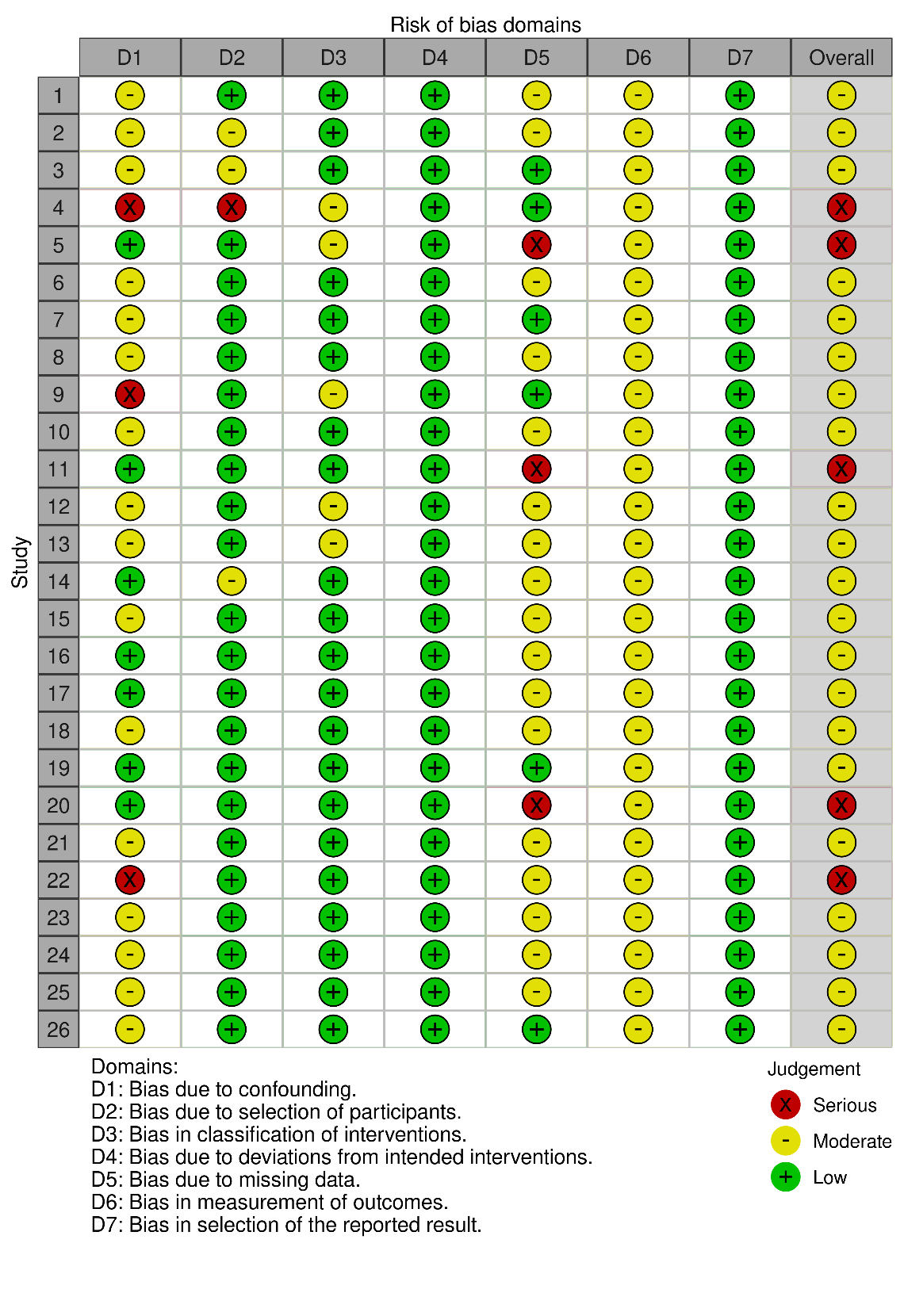


**Supplementary Figure 20.** The traffic light plot for the included prenatal studies


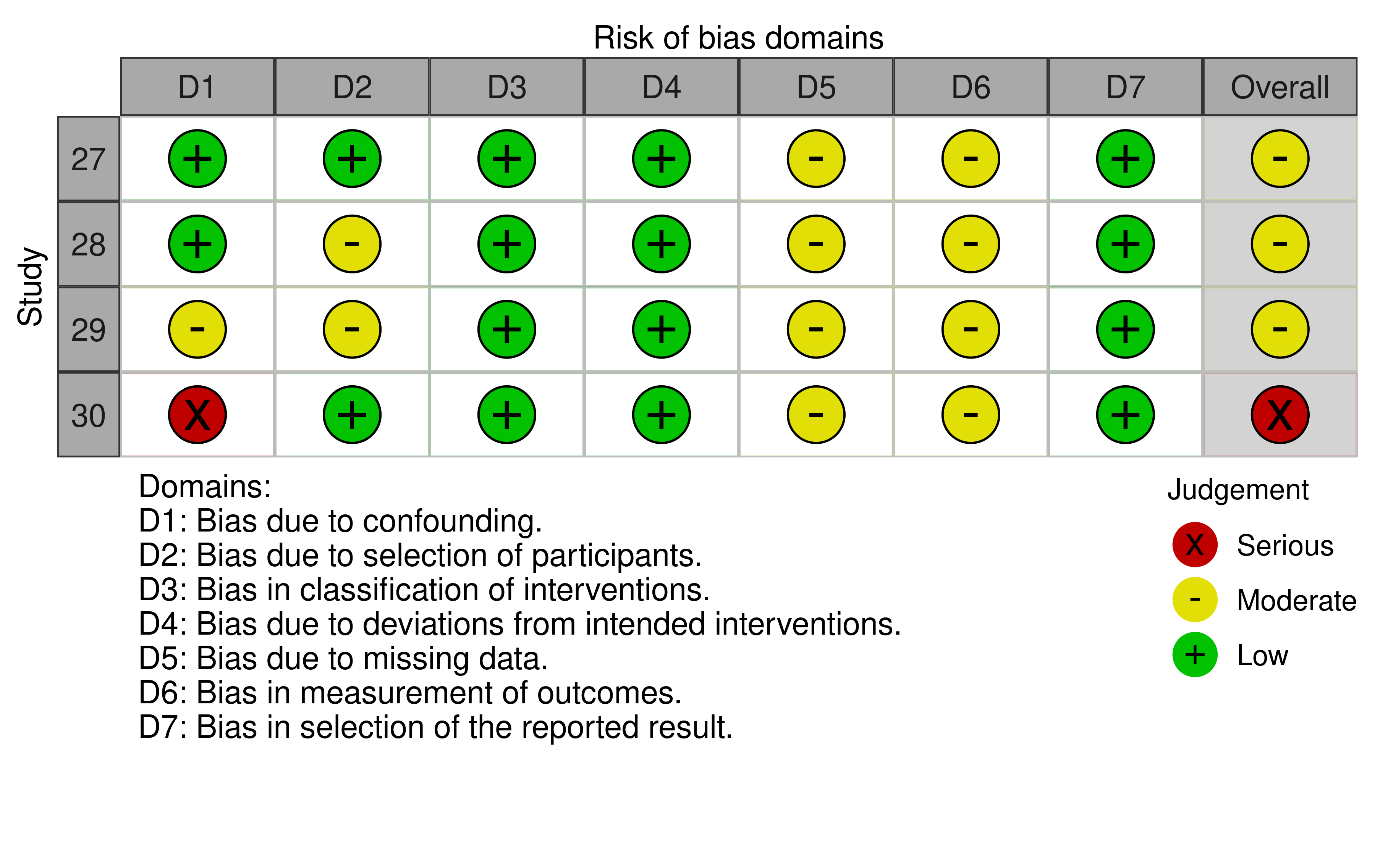


**Supplementary figure 21.** The traffic light plot for the included intrapartum studies
